# Exploring celiac disease candidate pathways by global gene expression profiling and gene network cluster analysis

**DOI:** 10.1038/s41598-020-73288-6

**Published:** 2020-10-01

**Authors:** Babajan Banaganapalli, Haifa Mansour, Arif Mohammed, Arwa Mastoor Alharthi, Nada Mohammed Aljuaid, Khalidah Khalid Nasser, Aftab Ahmad, Omar I. Saadah, Jumana Yousuf Al-Aama, Ramu Elango, Noor Ahmad Shaik

**Affiliations:** 1 Department of Genetic Medicine, Faculty of Medicine, King Abdulaziz University, Jeddah, 21589 Saudi Arabia; 2 Princess Al-Jawhara Al-Brahim Center of Excellence in Research of Hereditary Disorders, King Abdulaziz University, Jeddah, Saudi Arabia; 3 Department of Biology, College of Science, University of Jeddah, Jeddah, Saudi Arabia; 4 Department of Biological Sciences, Faculty of Science, King Abdulaziz University, Jeddah, Saudi Arabia; 5 Department of Medical Laboratory Technology, Faculty of Applied Medical Sciences, King Abdulaziz University, Jeddah, Saudi Arabia; 6 Department of Health Information Technology, Faculty of Applied Studies, King Abdulaziz University, Jeddah, Saudi Arabia; 7 Pediatric Gastroenterology Unit, Department of Pediatrics, Faculty of Medicine, King Abdulaziz University, Jeddah, Saudi Arabia

**Keywords:** Molecular biology, Computational biology and bioinformatics

## Abstract

Celiac disease (CeD) is a gastrointestinal autoimmune disorder, whose specific molecular basis is not yet fully interpreted. Therefore, in this study, we compared the global gene expression profile of duodenum tissues from CeD patients, both at the time of disease diagnosis and after two years of the gluten-free diet. A series of advanced systems biology approaches like differential gene expression, protein–protein interactions, gene network-cluster analysis were deployed to annotate the candidate pathways relevant to CeD pathogenesis. The duodenum tissues from CeD patients revealed the differential expression of 106 up- and 193 down-regulated genes. The pathway enrichment of differentially expressed genes (DEGs) highlights the involvement of biological pathways related to loss of cell division regulation (cell cycle, p53 signalling pathway), immune system processes (NOD-like receptor signalling pathway, Th1, and Th2 cell differentiation, IL-17 signalling pathway) and impaired metabolism and absorption (mineral and vitamin absorptions and drug metabolism) in celiac disease. The molecular dysfunctions of these 3 biological events tend to increase the number of intraepithelial lymphocytes (IELs) and villous atrophy of the duodenal mucosa promoting the development of CeD. For the first time, this study highlights the involvement of aberrant cell division, immune system, absorption, and metabolism pathways in CeD pathophysiology and presents potential novel therapeutic opportunities.

## Introduction

Celiac disease (CeD) is a gluten-induced autoimmune disease seen in genetically susceptible people^1^. It is estimated to be prevalent in 1% of the world population^2,3^. CeD patients exhibit severe gastrointestinal symptoms such as diarrhoea, bloating, and abdominal pain following gluten consumption which is commonly found in wheat, rye and barely^4^. Other manifestations of the disease involve malabsorption and anaemia, which are consequences of the villus atrophy in small intestine^4,5^. Adopting a gluten-free diet results in the clinical and histological improvements in patients. However, a substantial portion of the patients exhibit symptoms and persistent villus atrophy even after dietary management^6,7^. Patients with CeD demonstrate other autoimmune diseases such as type 1 diabetes, thyroid disease, multiple sclerosis and inflammatory bowel disease, more frequently (∼5%) than healthy individuals^8^. Several factors like genetic background, autoimmunity, environment (gluten as the main factor) and gut microbiome are mainly implicated in the etiology of CeD.

The genetic liability of CeD is supported by the involvement of both HLA (40%) and non-HLA genes (60%) in its etiology^9^. The HLA variants (DQA1 and DQB1), encode two antigens related to CeD, of which HLA-DQ2 antigen is found in 90% of CeD patients and is associated with stronger gluten-specific T helper cell response^10^. The second antigen HLA-DQ8 is found in the remaining patients. Interestingly, 30–40% of the general population carry these risk alleles but do not present any CeD symptoms when exposed to dietary gluten. This suggests that HLA-DQ2 or HLA-DQ8 alleles act as a prerequisite but not determine the development of CeD in individuals. Hence, non-HLA genes are assumed to play a critical role in the disease pathogenesis^11^. Early genome-wide association studies (GWAS) conducted on CeD have discovered that non-HLA genes like IL2 and IL21, which are involved in T cell maturation, can modulate the risk of disease development in genetically susceptible individuals^12–14^. Since then, several follow-up population genetics and *in-vitro* functional studies have also underlined the potential molecular crosstalk between HLA and non-HLA risk alleles, genetic expression and epigenetic changes, which subsequently triggers the cascade of autoimmune reactions critical to the development of CeD^15–18^.

The genetic etiology of CeD is so far widely studied by different genetic approaches like candidate gene sequencing, exome sequencing, SNP genotyping and epigenetic screening^16,19–22^. However, compared to the above-mentioned genotyping approaches, there are very few gene expression studies which have assessed the contribution of genes to the pathophysiology of CeD. Moreover, those gene expression studies have only used basic statistical methods to explore the up or down expressed genes. The noise and bias of gene expression measurements and regulation of gene expression at post transcription level pose an additional challenge to interpret the actual role of individual or group of genes in celiac disease. Therefore, combining the gene expression measurements with protein–protein interactions (PPIs) and pathway analysis will provide a deeper insight into gene expression induced CeD development.

Thus, we conducted this first systems biology study to compare the gene expression profile of duodenum tissue samples of celiac patients at diagnosis and after restricted gluten-free diet. This study characterized the protein interactions and molecular pathways involving several differentially expressed genes (DEGs) and provided a global view of gene expression changes critical to CeD pathogenesis, which presents potential therapeutic avenues for future research.

## Materials and methods

### Gene datasets sources

Gene expression changes in CeD patients were compared in different conditions; at disease diagnosis, post-gluten-free dietary management as well as after in-vitro gliadin challenge. The gene expression profiles from the above mentioned three conditions were downloaded from the public domain Array Express—functional genomics data (https://www.ebi.ac.uk/arrayexpress/). These gene expression profiles were generated on Affymetrix Human Genome U133 Plus 2.0 Array, GPL570 platform (Affymetrix, Santa Clara, CA USA). The full details about tissue processing, RNA isolation, hybridization of arrays can be found in the original research article^23^.

The gene expression profile of duodenum tissue biopsies after two years of gluten-free diet (n = 9, control samples) was compared to two different gluten exposure conditions. The first one is at disease diagnosis (chronic exposure, test samples, n = 9), and the corresponding dataset Array Express ID is E-MEXP-1828. The diagnosis was based on positive CeD-associated antibodies and a histological classification of intestinal villi was done according to Marsh staging grade 3b or c changes (villous atrophy). The second condition is in-vitro gliadin challenge (acute exposure, test samples, n = 9), and its corresponding dataset Array Express ID is E-1823^24^.

### Data processing

Preprocessing of gene expression data sets was performed using R package (https://www.r-project.org)^25^. To standardize and reduce the technical noise in the sample data, raw intensity signals in the CEL file format were loaded into the Bioconductor*-Affy* package and the raw signal values of each sample set were standardized to a median of all samples using the Robust Multiarray Average (RMA) algorithm by baseline^25,26^. This algorithm normalizes the raw signals by generating a matrix of expression from the data with context correction and log^2^ conversion followed by quantile normalization.

### DEGs screening

Limma package (https://bioconductor.org/packages/release/bioc/html/limma.html) was used to obtain the required tools to analyze DEGs with t-test^27^. False discovery rate (FDR) was calculated using Benjamini & Hochberg method^28^. The logFC cut off value for DEGs was |log FC|> 1.5, and the FDR was < 0.01 while p-value was < 0.05^29^. Heatmap was generated for each dataset using Heatmap online software (https://www.heatmapper.ca) to represent significant DEGs.

### PPI construction, cluster networks and hub genes identification

The DEGs were classified into up- and down-regulated genes and then analyzed in STRING database (https://string-db.org) for detecting differences in the PPI network^30^. The STRING selection is based on different parameters of direct and indirect interactions. Statistical information about each PPI network was obtained using STRING. The maximum PPI enrichment p-value was < 1.0 × 10^–16^ and the minimum average local clustering coefficient was > 0.4. Both Up- and down-regulated PPI networks were visualized using Cytoscape 3.7.1 software^31^. Molecular Complex Detection (MCODE) tool was used to screen out clusters of PPI networks with the following parameters, degree cutoff of 2, node score cutoff of 0.2, k‐core = 2, and max depth of 100^32^. Genes with the highest MCODE scores were identified as hub genes by Cytoscape plug-in cytoHubba.

### Functional annotations of cluster networks

Both up- and down-regulated (PPI networks and network clusters) genes were provided as an input to Cytoscape 3.7.1 software for recognizing GO terms and pathways using functional analysis modules of ClueGo and Cluepedia tools. GO annotations interpret the association of gene products to biological process (BP), molecular function (MF), cellular component (CC), Kyoto Encyclopedia of Genes and Genomes (KEGG) pathways^33,34^ (https://www.kegg.jp/kegg/kegg1.html) and immune system processes (ISP)^35–37^ . The selection criteria included a minimum of 3 genes in the cluster with GO tree interval range in between 3 and 8 and a kappa score of 0.4 for pathway network connectivity^38,39^. The Bonferroni step-down (pV correction) method with two-sided hypergeometric test option was selected for statistical assessment. With the aforementioned parameters we have chosen GO term fusion and restriction for creating ClueGO category network based on network overlapping at a statistical significance of P < 0.05.

## Results

### Data processing and DEGs screening

**Figure 1:**
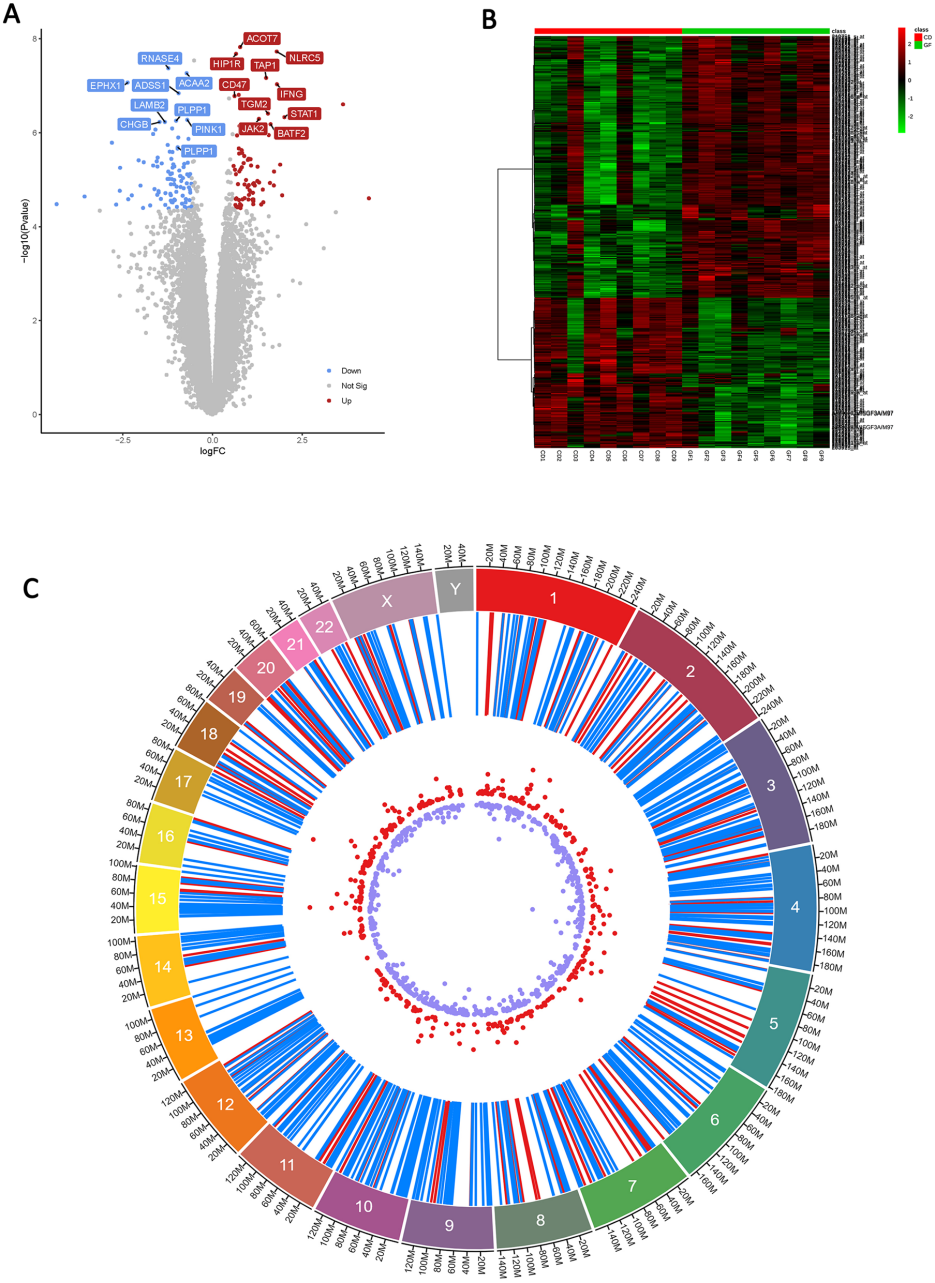
The differentially expressed genes (DEGs) analysis of duodenum tissue at the time of CeD diagnosis in comparison to gluten restricted dietary managament. (**A**) Volcano plots of Log fold change of gene expression. (**B**) Heatmap of the DEGs with a LogFC > 1.5. Red: up-regulation; green: down-regulation. (**C**) Circos view of localization of DEGs on chromosomes (first track-chromosome number, second track- DEGs, Third track Up (Red) and Down (Blue) genes) (Circos figure generated using: https://marianattestad.com/chordial).

The comparison of expression profiles between CeD at the time of diagnosis and after two years of gluten-free diet condition revealed the differential expression of 299 genes (corresponding to 425 probes), including 106 upregulated and 193 downregulated genes. Top five DEGs are presented in Table 1. Among the 106 upregulated genes, LPL has the highest LogFC of 4.36. Similarly, APOA1 has the lowest LogFC value of -4.34 among 193 downregulated genes. The volcano plot represents the log2FC and the heatmap shows the DEGs in all the samples (Fig. 1). On the contrary, gluten-free diet versus in-vitro gliadin challenge analysis showed that global gene expression changes were less than 1.5 folds and insignificant, hence they were omitted from further analysis. The significant DEGs (more than 1.5 folds) from at the time of diagnosis versus gluten-free diet groups were selected for further analysis (Supplementary data Figure S1).

**Table 1:** Top five differentially expressed genes (DEGs) in intestinal duodenum tissues at the time of CeD diagnosis versus post-gluten free dietary management.

Top 5 up and down regulated genes					
	Gene ID	LogFC	T-test	P-value	FDR
Upregulated	LPL	4.36342621	5.51	2.50 × 10^–5^	8.33 × 10^–5^
	CXCL11	3.44269906	5.20	4.95 × 10^–5^	8.25 × 10^–5^
	MMP3	3.10503802	4.42	2.9 × 10^–4^	3.62 × 10^–4^
	LCN2	2.61525006	4.94	8.92 × 10^–5^	1.78 × 10^–4^
	MMP12	2.44493598	3.67	1.61 × 10^–3^	3.22 × 10–3
Downregulated	APOA1	−4.3448706	−5.38	3.33 × 10^–5^	8.32 × 10^–5^
	HMGCS2	−3.5650963	−5.55	2.29 × 10^–5^	1.14 × 10^–4^
	CYP3A4	−3.1418	−5.23	4.59 × 10^–5^	9.18 × 10^–5^
	DGAT2	−2.803011	−6.80	1.64 × 10^–6^	1.64 × 10^–5^
	APOC3	−2.7603543	−4.35	3.36 × 10^–4^	3.74 × 10^–4^

### PPI networks of up and down regulated genes

**Figure 2:**
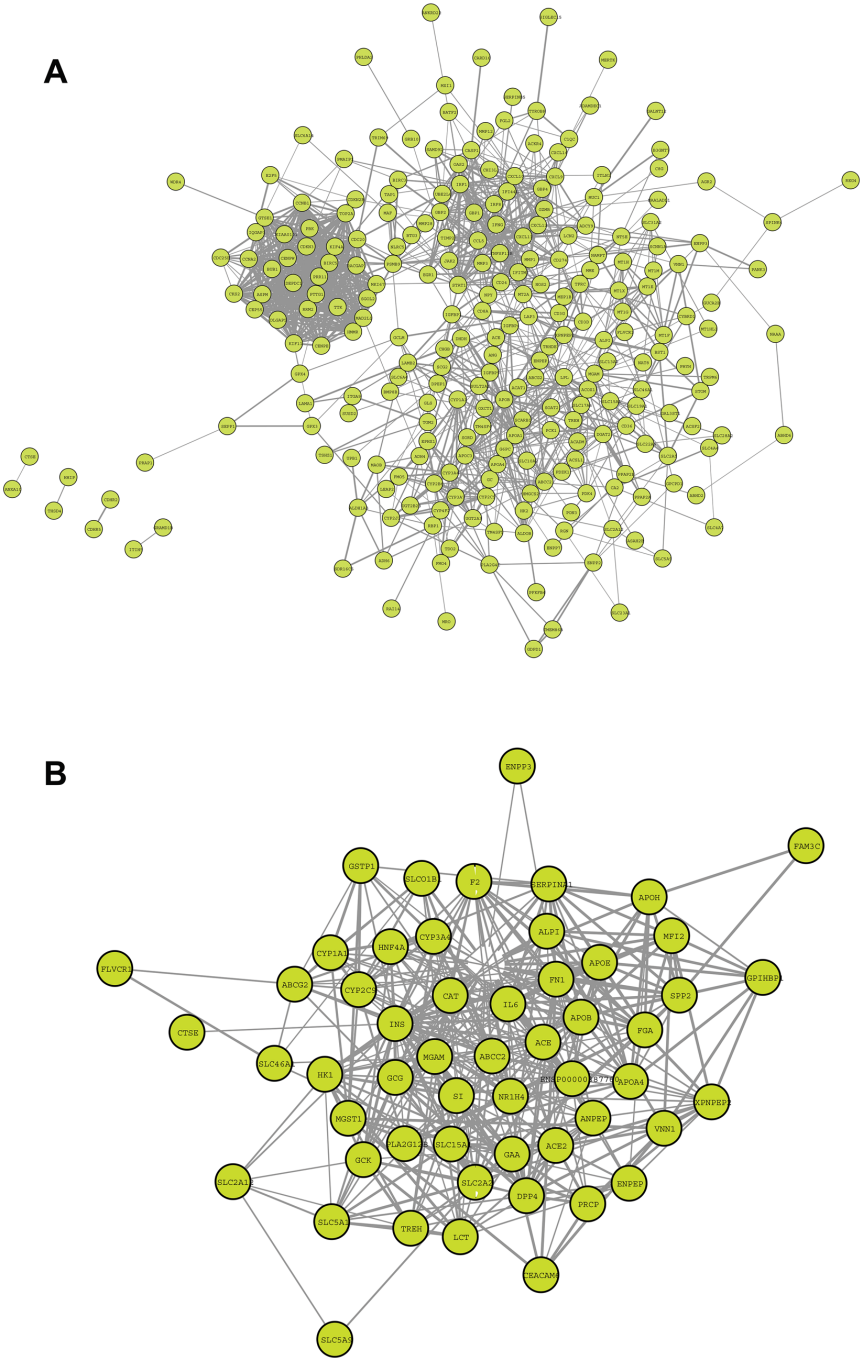
Overview of PPI network constructed using Cytoscape STRING database. (**A**) upregulated (**B**) down regulated PPI network, the density of the network nodes is based on string confidence score > 0.7 (Network Figures generated using https://cytoscape.org/).

PPI networks highlight the physical contacts among protein partners. They are critical in most basic molecular mechanisms involved in cellular function but are often perturbed in disease states. The PPI networks of upregulated DEGs included 103 nodes connecting 664 edges with a clustering coefficient of 0.531 and network centralization of 0.221. While the downregulated PPIs included 188 nodes connecting 444 edges with a clustering coefficient of 0.256 and network centralization of 0.120 (Fig. 2).

The gene ontology analysis of upregulated DEGs showed their significant enrichment in two broad groups namely cell cycle regulation and immune system function, under biological processes ontology source (Figures S2, S3). Gene expression changes in cell components were mainly enriched in the spindle, midbody, condensed chromosome kinetochore, and centromeric region, which are involved in cytokinesis processes at the end of cell division (Supplementary data Figure S4). In molecular function annotation, gene expression alterations were associated with regulation of enzyme activities of endopeptidase, peptidase, and cysteine-type endopeptidase, which are mainly involved in activating cell-mediated immunity, autoimmune and inflammatory responses (Supplementary data Figure S5). The KEGG analysis revealed that DEGs were connected to cell cycle, p53 signalling pathway and apoptosis, where dysfunction of p53 and apoptosis are known for their association with autoimmunity^33,34^ (Supplementary data Figure S6). Further classification of all upregulated DEGs under GO ontology source revealed their significant enrichment in immune system processes. Their pathway enrichment analysis showed that response to interferon-gamma, regulation of T-cell proliferation, antigen processing, presentation of exogenous peptide antigens, NOD-like receptor signalling, Th1 and Th2 cell differentiation, IL-17 signalling pathway were branch end terms (Fig. 2 and Supplementary data Tables S1, S2).

**Figure 3:**
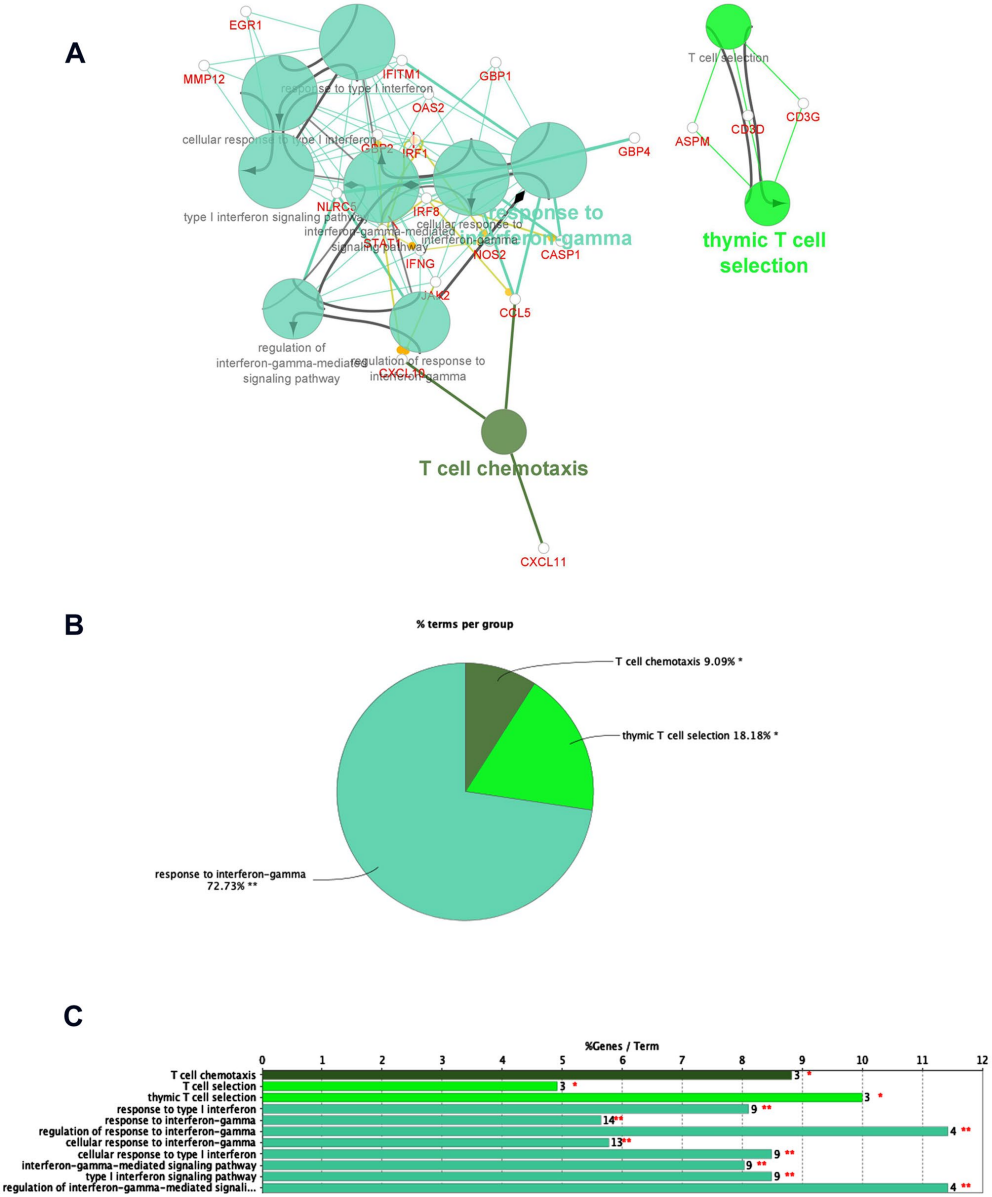
Enriched Immune system groups using the ClueGo and CluePedia plugins of Cytoscape. (**A**) GO/immune pathwy terms specific for upregulated genes. (**B**) An overview pie chart with functional groups, including specific terms for the upregulated proteins in the immune pathways. (**C**) The bars represent the number of genes associated with the immune pathway (**A**–**D** Figures generated using https://cytoscape.org/).

**Figure 4:**
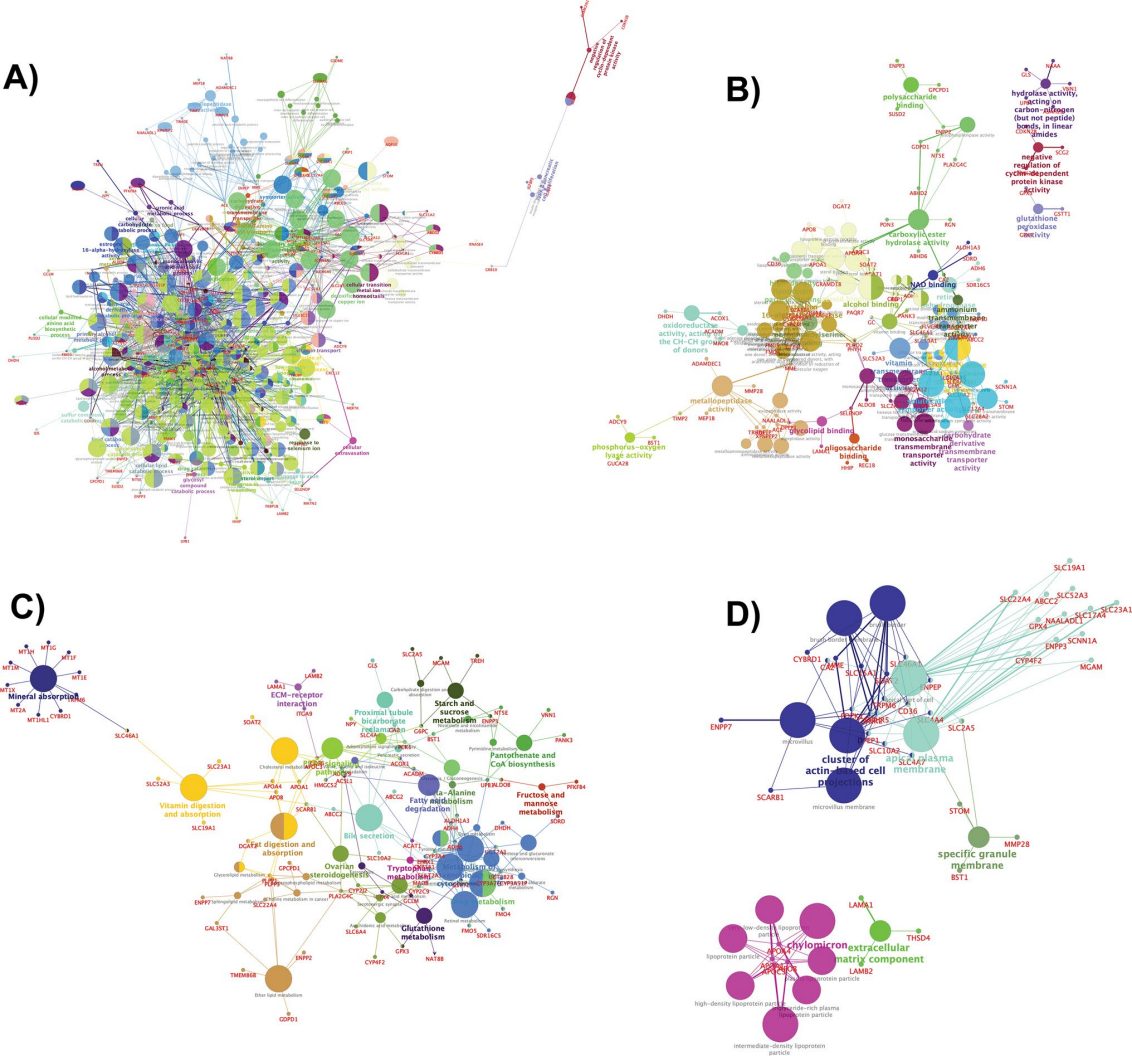
ClueGO analysis of the predicted Go Annotations. Functionally grouped network of enriched categories was generated for the target genes. GO terms are represented as nodes, and the node size represents the term enrichment significance. (**A**) Representative Biological Process (**B**) Molecular Function (**C**) KEGG Pathways (**D**) Cellular components interactions among predicted targets. (**A**–**D** Figures generated using https://cytoscape.org/).

GO analysis of down-regulated DEGs showed their relation to metabolic and transport processes of a variety of molecules (Fig. 3). Some BP annotations include cellular lipid catabolism processes involved in lipid breakdown, and detoxification of inorganic compounds (Supplementary data Figure S7). MF annotations include symporter activity, which enables active transporting across the membrane and secondary active transmembrane transporter activity, which is a wider term involving solute transportation across the membrane (Supplementary data table S3 and Figure S8). The CC annotations included apical plasma membrane which is the microvilli surface of the lumen and cluster of actin-based cell projections, which form the microvilli of the small intestine. KEGG pathways highlighted mineral absorption, drug metabolism, vitamin digestion and absorption (Fig. 4 and Supplementary data S9, S10).

### Cluster networks and hub genes identification using MCODE scores

**Figure 5:**
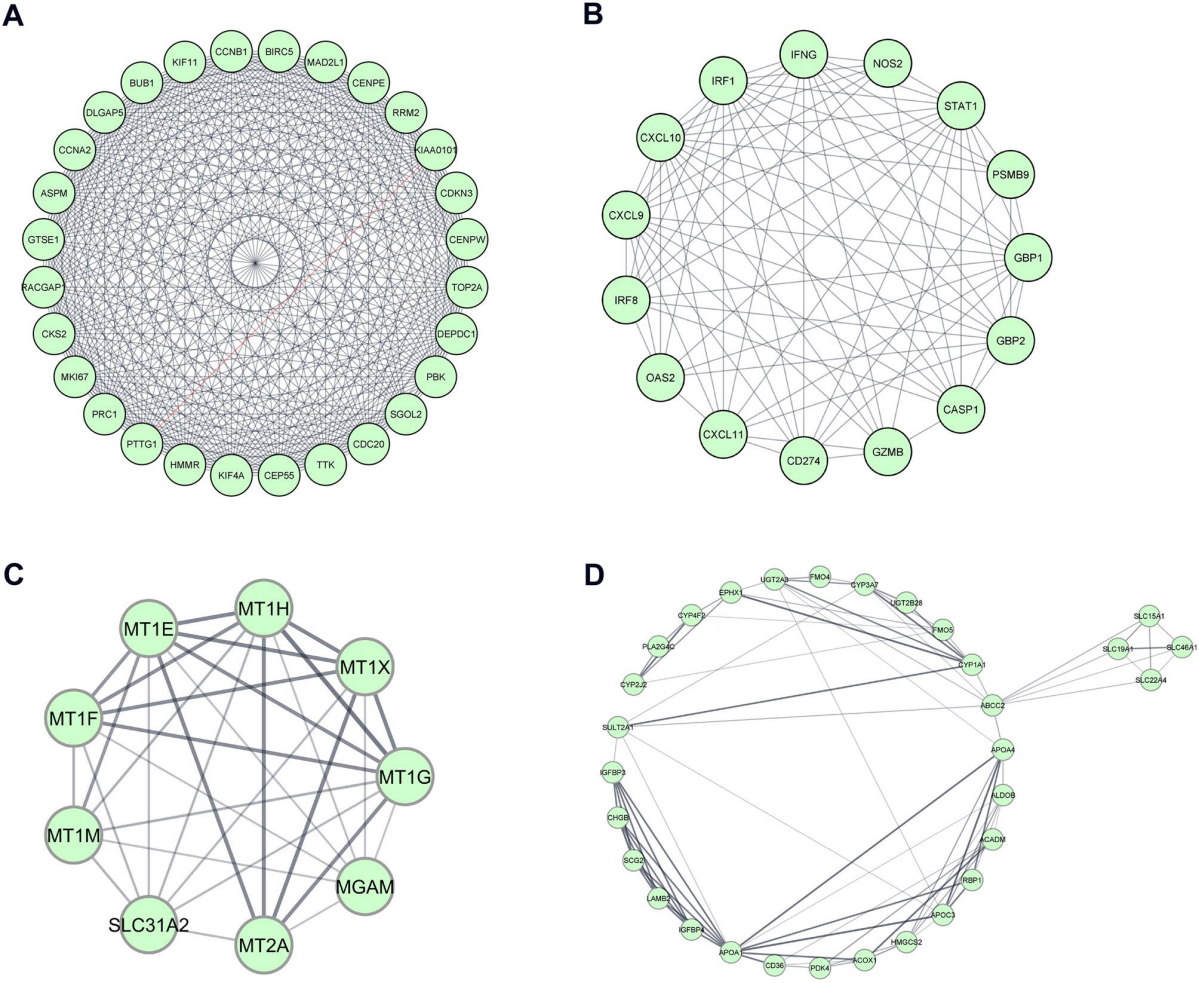
The MCODE clusters and hub genes identified from DEGs in duodenum tissue of celiac patients. Upregulated (**A**) Cluster-1. (**B**) Cluster-2, and Downregulated (**C**) Cluster-1. (**D**) Cluster-2 classified based on MCC score > 5. (A-D Figures generated using https://cytoscape.org/).

Protein interaction network clusters are a group of proteins with great functional similarity than proteins in different clusters, whereas hub genes are functionally significant interconnected nodes in a cluster. MCODE is a Cytoscape plugin that searches for clusters (highly interconnected regions) in a protein interaction network. The PPI network analysis of up and down-regulated DEGs revealed two significant cluster networks from each category (MCODE score of > 5). From the upregulated PPI network, cluster 1, showed 28 nodes linked via 365 edges with an MCODE score of 27.037. The top nodes in this cluster showing MCODE scores of > 23 (PTTG1, CDC20, TTK, BIRC5 and DEPDC1) were identified as hub genes for CeD. The cluster 2 shows 15 nodes linked via 80 edges with MCODE score of 11.429. In cluster 2, the top 4 genes (CXCL9, CXCL10, IRF1 and STAT1) with MCODE scores > 7 were identified as hub genes for CeD. For the downregulated PPI network, the cluster 1, shows 9 nodes linked via 32 edges; of which 5 (55.5%) were identified as hubs with MCODE score of 5.8. The top 3 hub genes (MT1H, MT1G and MT1E) identified for CeD from this cluster had an MCODE score of > 5.2. The second cluster has an MCODE score of 5.4 and is characterized by 31 nodes linked to 81 edges (Fig. 5). The top 2 hub genes showing an MCODE score of > 6 from this cluster were IGFBP3 and APOA1.

### GO annotations of network clusters

The top cluster networks from MCODE were used as input for analyzing the PPI functional enrichment maps using ClueGo and CluePedia plugins. Tables 2 and 3 shows, highly significant GO annotation clusters with an p-value of < 1.35 × 10^–2^. The cluster 1 from upregulated DEGs network in BP ontology source has projected mitotic nuclear division and sister chromatid segregation as top GO terms. In MF ontology source, the top GO term was cyclin-dependent protein serine/threonine kinase regulator activity. For CC ontology source, the significant GO terms were related to kinetochore and spindle microtubule. KEGG pathway ontology source included cell cycle and p53 signalling pathway as significant GO terms, whereas cluster-2 was related to immune system processes. From BP ontology source, the top GO terms were cellular response to interferon-gamma and its interferon-gamma signalling pathway. These two GO terms were also seen to be significant under ISP ontology source. MF ontology source highlighted CXCR chemokine receptor binding especially CXCR3 as top GO terms.

**Table 2:** Functional enrichment of MCODE cluster networks of upregulated DEGs highlights GO terms related to cell division and immune system.

Upregulated DEG clusters	Ontology source	Term ID	GO term	Term P value	FDR
Cluster-1	Biological Processes (BP)	GO:0,140,014	mitotic nuclear division	1.80 × 10^–20^	9.02 × 10^–20^
		GO:0,000,819	sister chromatid segregation	1.17 × 10^–17^	2.92 × 10^–17^
		GO:0,007,088	regulation of mitotic nuclear division	9.77 × 10^–16^	1.63 × 10^–15^
		GO:0,000,070	mitotic sister chromatid segregation	3.72 × 10^–15^	2.24 × 10^–14^
		GO:0,051,783	regulation of nuclear division	4.32 × 10^–15^	1.30 × 10^–14^
	Molecular Functions (MF)	GO:0,016,538	cyclin-dependent protein serine/threonine kinase regulator activity	1.70 × 10^–4^	2.13 × 10^–4^
	Cellular Components (CC)	GO:0,000,776	Kinetochore	1.07 × 10^–10^	1.80 × 10^–10^
		GO:0,000,777	condensed chromosome kinetochore	3.88 × 10^–10^	1.36 × 10^–9^
		GO:0,000,779	condensed chromosome, centromeric region	1.12 × 10^–9^	2.63 × 10^–9^
		GO:0,005,876	spindle microtubule	7.48 × 10^–8^	9.36 × 10^–8^
		GO:0,000,307	cyclin-dependent protein kinase holoenzyme complex	7.84 × 10^–5^	1.09 × 10^–4^
	KEGG Pathways (KP)	KEGG:04,110	Cell cycle	2.08 × 10^–10^	3.47 × 10^–10^
		KEGG:04,114	Oocyte meiosis	8.49 × 10^–7^	1.70 × 10–6
		KEGG:04,914	Progesterone-mediated oocyte maturation	1.29 × 10^–5^	2.07 × 10^–5^
		KEGG:04,115	p53 signaling pathway	1.74 × 10^–4^	1.74 × 10^–4^
Cluster-2	Biological Processes (BP)	GO:0,071,346	cellular response to interferon-gamma	2.84 × 10^–14^	1.42 × 10^–13^
		GO:0,060,333	interferon-gamma-mediated signaling pathway	1.78 × 10^–12^	4.45 × 10^–12^
		GO:0,071,357	cellular response to type I interferon	1.80 × 10^–8^	2.52 × 10^–8^
		GO:0,060,337	type I interferon signaling pathway	1.80 × 10^–8^	2.52 × 10^–8^
		GO:0,034,340	response to type I interferon	2.27 × 10^–8^	6.81 × 10^–8^
	Molecular Functions (MF)	GO:0,048,248	CXCR3 chemokine receptor binding	9.28 × 10^–9^	4.64 × 10^–8^
		GO:0,045,236	CXCR chemokine receptor binding	1.32 × 10^–7^	2.20 × 10^–7^
		GO:0,042,379	chemokine receptor binding	7.92 × 10^–7^	1.43 × 10^–6^
		GO:0,008,009	chemokine activity	1.96 × 10^–5^	2.53 × 10^–5^
	KEGG Pathways (KP)	KEGG:05,133	Pertussis	1.21 × 10^–5^	1.52 × 10^–5^
		KEGG:05,140	Leishmaniasis	3.83 × 10^–4^	3.83 × 10^–4^
	Immune System Processes (ISP)	GO:0,071,346	cellular response to interferon-gamma	2.30 × 10^–8^	5.77 × 10^–8^
		GO:0,060,333	interferon-gamma-mediated signaling pathway	7.28 × 10^–8^	9.11 × 10^–8^
		GO:0,071,357	cellular response to type I interferon	3.31 × 10^–5^	6.63 × 10^–5^
		GO:0,060,337	type I interferon signaling pathway	3.31 × 10^–5^	6.63 × 10^–5^
		GO:0,034,340	response to type I interferon	4.15 × 10^–5^	4.98 × 10^–5^

**Table 3:** Functional enrichment of MCODE cluster networks of downregulated DEGs highlights GO terms related to mineral absorption and metabolism.

Down-regulated DEG modules	Ontology Source	Term ID	GO Term	Term PValue	FDR
Cluster-1	Biological Processes (BP)	GO:1,990,169	stress response to copper ion	9.10 × 10^–21^	1.00 × 10^–19^
		GO:0,061,687	detoxification of inorganic compound	1.44 × 10^–20^	7.21 × 10^–20^
		GO:0,097,501	stress response to metal ion	2.21 × 10^–20^	5.55 × 10^–20^
		GO:0,071,294	cellular response to zinc ion	1.88 × 10^–19^	5.17 × 10^–19^
		GO:0,071,276	cellular response to cadmium ion	1.09 × 10^–17^	2.41 × 10^–17^
	KEGG Pathways (KP)	KEGG:04,978	Mineral absorption	3.45 × 10^–15^	5.75 × 10^–15^
Cluster-2	Biological Processes (BP)	GO:0,006,721	terpenoid metabolic process	1.82 × 10^–7^	1.82 × 10^–7^
		GO:0,050,892	intestinal absorption	1.30 × 10^–6^	4.56 × 10^–6^
		GO:0,035,376	sterol import	1.64 × 10^–6^	2.00 × 10^–6^
		GO:0,070,508	cholesterol import	1.63 × 10^–6^	1.80 × 10^–6^
		GO:0,034,371	chylomicron remodeling	2.04 × 10^–6^	1.84 × 10^–5^
	Molecular Functions (MF)	GO:0,072,349	modified amino acid transmembrane transporter activity	2.20 × 10^–5^	3.08 × 10^–5^
		GO:0,050,660	flavin adenine dinucleotide binding	2.39 × 10^–5^	4.31 × 10^–5^
		GO:0,016,712	oxidoreductase activity, acting on paired donors, with incorporation or reduction of molecular oxygen, reduced flavin or flavoprotein as one donor, and incorporation of one atom of oxygen	2.89 × 10^–5^	4.34 × 10^–5^
		GO:0,008,395	steroid hydroxylase activity	3.42 × 10^–5^	4.41 × 10^–5^
		GO:0,005,310	dicarboxylic acid transmembrane transporter activity	1.26 × 10^–4^	1.26 × 10^–4^
	Cellular Components (CC)	GO:0,042,627	Chylomicron	3.69 × 10^–6^	8.61 × 10^–6^
		GO:0,034,385	triglyceride-rich plasma lipoprotein particle	1.10 × 10^–5^	1.84 × 10^–5^
		GO:0,034,361	very-low-density lipoprotein particle	1.10 × 10^–5^	1.93 × 10^–5^
		GO:0,034,364	high-density lipoprotein particle	2.90 × 10^–5^	4.84 × 10^–5^
		GO:0,034,358	plasma lipoprotein particle	7.66 × 10^–5^	9.58 × 10^–5^
	KEGG Pathways (KP)	KEGG:03,320	PPAR signaling pathway	1.46 × 10^–7^	1.17 × 10^–6^
		KEGG:05,204	Chemical carcinogenesis	2.72 × 10^–7^	1.09 × 10^–6^
		KEGG:04,977	Vitamin digestion and absorption	1.14 × 10^–6^	8.00 × 10^–6^
		KEGG:00,980	Metabolism of xenobiotics by cytochrome P450	5.17 × 10^–6^	1.03 × 10^–5^
		KEGG:04,979	Cholesterol metabolism	2.33 × 10^–5^	2.71 × 10^–5^

Cluster-1 of downregulated DEGs showed that the genes in this cluster were particularly related to mineral absorption and detoxification. The BP ontology source highlighted the detoxification of inorganic compound and stress response to metal ions as top GO terms. While the KEGG ontology source identified mineral absorption pathway as the significant GO term. The cluster-2 (from downregulated DEGs) was related to metabolism and absorption of diverse sets of molecules. BP highlighted GO terms like terpenoid metabolic process which is an organic compound and intestinal absorption. MF ontology source showed modified amino acid transmembrane transporter activity and dicarboxylic acid transmembrane transporter activity as top GO terms. CC ontology source has highlighted lipid absorption and metabolism-related GO annotations including chylomicron which are responsible for lipid transport and very-low-density lipoprotein particle. KEGG underlined GO terms like vitamin digestion and absorption as well as cholesterol metabolism.

## Discussion

CeD is a complex multifactorial enteropathy where transglutaminase-deamidated gliadin peptides act as just initial event, but the actual anatomical and histological presentation of the disease is determined by multiple genomic and proteomic alterations taking place in a complex biological network^24^. Thus, global gene expression, which involves studying expression changes in both immune response genes as well as non-immune response genes controlling the gliadin peptide recognition is an attractive strategy to identify the potential molecular pathological networks involved in CeD development. Several gene expression studies have investigated biological pathways essential for the development of CeD in intestinal tissues^40,41^ and specific cell types^42^. By integrating gene expression data with protein interaction network concepts, this study has identified the contribution of dysregulated immune system genes in the intestinal mucosa of CeD. Furthermore, this study reports that gene expression alterations in pathways connected to cell division regulation may have a compensatory role to contain the intestinal mucosal injury due to prolonged autoimmune responses. The additional noteworthy finding is related to impeded absorption, metabolism, and transportation of mineral and vitamins in the intestinal tissues, which eventually increases the likelihood of malnutrition alongside the role of villus atrophy in CeD^43^.

GO annotations interpret the association of gene products to certain pathways from published works  on disease etiology and development ^44^. Majority of the annotations are enriched in the up- and down-regulated PPI clusters represent the most interacting group of genes amongst the whole PPI networks; especially, hub genes, which showed highest connectivity and correlation to their modules. Diverse pathways of hub genes connected to dysregulation of the immune system in intestinal duodenum tissues were enriched in the overexpressed genes and subsequently in PPI networks and its functional clusters. In the upregulated DEGs, KEGG pathway (https://www.kegg.jp/kegg/kegg1.html) identified the significant enrichment of signalling pathways like NOD-like receptors (NLRs) and Toll-like receptors (TLRs). Both NOD-like and Toll-like receptors take part in mediating immune recognition by initiating innate immunity and activating adaptive immunity. Specifically, NLRP3 inflammasome (a member of NLRs family) is associated with innate immunity in response to the wheat protein in CeD knockout mice^45^. Other enriched pathways included genes controlling TNF and IL-17 signalling responses, as well as Th1, Th2 and Th17 differentiation. CD4 + T cells differentiation is directly correlated to autoimmunity, and it is induced by IFNγ and other cytokines including IL-17 and TNF protein^46^. This differentiation is essential for cytotoxic T lymphocyte activation, leading to intestinal epithelial cell destruction and villus atrophy^47^.

GO annotations of the immune-related module included signalling pathway of interferon-gamma (IFNγ), a major proinflammatory cytokine implicated in CeD, is well known for its role in regulating immune responses to infections and autoimmune diseases. IFNγ is also known to be very essential for the development of histopathological changes like villus atrophy, crypt hyperplasia in intestinal mucosa and production of CeD-associated antibodies, which mounts a strong adaptive immune response to develop CeD^47^. The additional key pathway enriched is chemokine signalling PPI cluster, which consists of CXCL9, CXCL10 and CXCL11 as hub genes. Another important hub gene from the immune-related module is STAT1, which is a direct activator of IFN-stimulated cells^48^. STAT1 has been previously associated with type 1 diabetes, which is caused by pancreatic β-cells destruction via cytokine-mediated apoptosis. Moreover, JAK2 gene, one of the gene from our upregulated PPI network, was previously reported to be overexpressed in intestinal tissues of adults and children CeD patients^49^. JAK2 is also critical for interleukin 12 (IL-12) signalling, whose production is attributed to several hub genes of this module such as interferon regulatory factors genes (IRF1, IRF8 and IFNG). Both IFNG and IL-12 contribute to T-helper1 cell differentiation and pathogenesis of systemic lupus erythematosus^50^. This suggests that dysregulated JAK-STAT cytokine signaling pathway mediates cascade of autoimmune reactions in CeD and other co-autoimmune conditions^51^.

Another major finding from upregulated cluster through KEGG pathway enrichment analysis includes cell cycle and p53 signalling pathways^33,34^, both of which are known play key role in the activation of intestinal mucosal cellular division and apoptosis^52^. The hub gene GTSE1 negatively regulates the p53 activity, hence it controls the downstream effects of p53 signalling pathway mediated cell cycle^53^. The Cyclin B2 (CCNA2) hub gene is directly involved in G2/M transition phase during the cell cycle and delays the cellular senescence and apoptosis by p53^54^. Other upregulated pathways reported in dietary gluten restricted mouse model of CeD are apoptosis and DNA repair in lamina propria and epithelium of the small intestine^47^. Upregulation of cell division related processes is thought to be a compensatory mechanism to the continuous apoptosis. The persistent apoptosis without sufficient cellular regeneration, causes villus atrophy of intestinal tissues, subsequently leading to malabsorption, a known complication in CeD patients^55^. The increased cellular division and abnormal activation of the immune system findings derived from the annotations of the upregulated PPI network and its clusters are consistent with the results of previous gene expression studies on CeD^24,56^.

The downregulated PPI network cluster results highlights the contribution of impaired homeostasis, digestion, metabolism and absorption pathways in CeD. Of these network clusters, mineral absorption pathway alterations including iron, copper, magnesium and zinc deficiencies are common clinical manifestations seen in CeD patients ^57^. This is finding is supported by the identification of the metallothionein genes as hub genes in the first downregulated clusters, which are involved in heavy metal homeostasis^58^. Another identified pathway is vitamin digestion and absorption, enriched by the SLC19A1, SLC46A, and other hub genes in the second downregulated module. Downregulation of this pathway could explain a common CeD clinical symptom- the multivitamin deficiency^57^. Along with the impaired vitamin absorption, folate (B9) is mainly absorbed in the duodenum, which is affected by villous atrophy, making the CeD patients five times more susceptible to folate deficiency than normal individuals. Lastly, cholesterol metabolism, fat digestion and absorption pathways are enriched in downregulated hub genes like APOA1, APOA4, and CD36. APOA1 is the major component of high-density lipoprotein (HDL) which is strongly associated with coronary heart disease (CHD)^59,60^. Both low HDL levels and risk of CHD have been reported in CeD patients^61^. GO annotations of the second cluster includes drug metabolism, metal ion homeostasis, lipid and other molecules transportation. Heme, bile acid and xenobiotic metabolism are downregulated in dietary gluten restricted mouse model of CeD^47^.

## Conclusion

This study highlights the utility of diverse system biology approaches for studying the gene expression profile of duodenum tissues to gain a comprehensive understanding about the underlying molecular mechanisms of CeD. Key pathways connected to potential biological events like (a) dysregulated immune system processes (NOD-like receptor signalling pathway, Th1 and Th2 cell differentiation, IL-17 signalling pathway), (b) loss of regulated cell division (cell cycle, p53 signalling pathway) and (c) impaired absorption (mineral and vitamin digestion and absorption as well as drug metabolism) were identified through protein interaction networks. All those pathways are connected to an increased number of intraepithelial lymphocytes (IELs) and villous atrophy of the duodenal mucosa. Validation of these biological pathways through functional studies could further confirm the present study findings. Furthermore, functional studies can then be utilized to identify the sensitive biomarker panel for diagnosis, prognosis, and novel drug targets for CeD.

## Supplementary information

Supplementary information.

## References

[1] Al-Bawardy B (2017). Celiac disease: a clinical review. Abdom. Radiol. (NY).

[2] Choung RS (2017). Prevalence and morbidity of undiagnosed celiac disease from a community-based study. Gastroenterology.

[3] Lebwohl B, Sanders DS, Green PHR (2018). Coeliac disease. Lancet (London, England).

[4] Mahadev S (2017). Factors associated with villus atrophy in symptomatic coeliac disease patients on a gluten-free diet. Aliment. Pharmacol. Ther..

[5] Lebwohl B, Murray JA, Rubio-Tapia A, Green PH, Ludvigsson JF (2014). Predictors of persistent villous atrophy in coeliac disease: a population-based study. Aliment. Pharmacol. Ther..

[6] Leffler DA (2007). Etiologies and predictors of diagnosis in nonresponsive celiac disease. Clin. Gastroenterol. Hepatol..

[7] Murray JA, Watson T, Clearman B, Mitros F (2004). Effect of a gluten-free diet on gastrointestinal symptoms in celiac disease. Am. J. Clin. Nutr..

[8] Lundin KE, Wijmenga C (2015). Coeliac disease and autoimmune disease-genetic overlap and screening. Nat. Rev. Gastroenterol. Hepatol..

[9] Romanos J (2009). Analysis of HLA and non-HLA alleles can identify individuals at high risk for celiac disease. Gastroenterology.

[10] Farina F (2019). HLA-DQA1 and HLA-DQB1 alleles, conferring susceptibility to celiac disease and type 1 diabetes, are more expressed than non-predisposing alleles and are coordinately regulated. Cells.

[11] Sharma A (2016). Identification of non-HLA genes associated with celiac disease and country-specific differences in a large, International Pediatric Cohort. PLoS ONE.

[12] van Heel DA (2007). A genome-wide association study for celiac disease identifies risk variants in the region harboring IL2 and IL21. Nat. Genetics.

[13] Saadah OI (2015). Replication of GWAS coding SNPs implicates MMEL1 as a potential susceptibility locus among Saudi Arabian celiac disease patients. Dis. Markers.

[14] Banaganapalli B (2017). Comprehensive computational analysis of GWAS loci identifies CCR2 as a candidate gene for celiac disease pathogenesis. J. Cell Biochem..

[15] Amr KS, Bayoumi FS, Eissa E, Abu-Zekry M (2019). Circulating microRNAs as potential non-invasive biomarkers in pediatric patients with celiac disease. Eur. Ann. Allergy Clin. Immunol..

[16] Perry AS, Baird AM, Gray SG (2015). Epigenetic methodologies for the study of celiac disease. Methods Mol. Biol. (Clifton NJ).

[17] Serena G, Lima R, Fasano A (2019). Genetic and environmental contributors for celiac disease. Curr. Allergy Asthma Rep..

[18] Khalesi M (2016). In vitro gluten challenge test for celiac disease diagnosis. J. Pediatr. Gastroenterol. Nutr..

[19] Trynka G (2011). Dense genotyping identifies and localizes multiple common and rare variant association signals in celiac disease. Nat. Genet..

[20] Fernandez-Jimenez N (2019). The methylome of the celiac intestinal epithelium harbours genotype-independent alterations in the HLA region. Sci. Rep..

[21] Al-Aama JY (2017). Whole exome sequencing of a consanguineous family identifies the possible modifying effect of a globally rare AK5 allelic variant in celiac disease development among Saudi patients. PLoS ONE.

[22] Szperl AM (2011). Exome sequencing in a family segregating for celiac disease. Clin. Genet..

[23] Jiang N (2008). Methods for evaluating gene expression from Affymetrix microarray datasets. BMC Bioinform..

[24] Castellanos-Rubio A (2010). Long-term and acute effects of gliadin on small intestine of patients on potentially pathogenic networks in celiac disease. Autoimmunity.

[25] Irizarry RA (2003). Exploration, normalization, and summaries of high density oligonucleotide array probe level data. Biostatistics.

[26] Gautier L, Cope L, Bolstad BM, Irizarry RA (2004). affy–analysis of Affymetrix GeneChip data at the probe level. Bioinformatics.

[27] Shi K, Li N, Yang M, Li W (2019). Identification of key genes and pathways in female lung cancer patients who never smoked by a bioinformatics analysis. J. Cancer.

[28] Benjamini Y, Hochberg Y (1995). Controlling the false discovery rate: a practical and powerful approach to multiple testing. J. R. Stat. Soc. Ser. B (Methodol.).

[29] Gentleman RC (2004). Bioconductor: open software development for computational biology and bioinformatics. Genome Biol..

[30] Szklarczyk D (2017). The STRING database in 2017: quality-controlled protein-protein association networks, made broadly accessible. Nucleic Acids Res..

[31] Sang L, Wang X-M, Xu D-Y, Zhao W-J (2018). Bioinformatics analysis of aberrantly methylated-differentially expressed genes and pathways in hepatocellular carcinoma. World J. Gastroenterol..

[32] Bader GD, Hogue CW (2003). An automated method for finding molecular complexes in large protein interaction networks. BMC Bioinform..

[33] Kanehisa M, Sato Y, Kawashima M, Furumichi M, Tanabe M (2016). KEGG as a reference resource for gene and protein annotation. Nucleic Acids Res..

[34] Kanehisa M, Goto S (2000). KEGG: kyoto encyclopedia of genes and genomes. Nucleic Acids Res..

[35] Sabir JSM (2020). Unraveling the role of salt-sensitivity genes in obesity with integrated network biology and co-expression analysis. PLoS ONE.

[36] Sabir JSM (2019). Identification of key regulatory genes connected to NF-kappaB family of proteins in visceral adipose tissues using gene expression and weighted protein interaction network. PLoS ONE.

[37] Sabir JSM (2019). Dissecting the Role of NF-kappab protein family and its regulators in rheumatoid arthritis using weighted gene co-expression network. Front. Genet..

[38] Bindea GMB, Hackl H, Charoentong P, Tosolini M (2009). ClueGO: a Cytoscape plug-in to decipher functionally grouped gene ontology and pathway annotation networks. Bioinformatics.

[39] Bindea GGJ, Mlecnik B (2013). CluePedia Cytoscape plugin: pathway insights using integrated experimental and in silico data. Bioinformatics.

[40] Fernandez-Jimenez N (2014). Coregulation and modulation of NFkappaB-related genes in celiac disease: uncovered aspects of gut mucosal inflammation. Hum. Mol. Genet..

[41] Bragde H, Jansson U, Fredrikson M, Grodzinsky E, Soderman J (2018). Celiac disease biomarkers identified by transcriptome analysis of small intestinal biopsies. Cell Mol. Life Sci..

[42] Quinn EM (2015). Transcriptome analysis of CD4+ T cells in coeliac disease reveals imprint of BACH2 and IFNgamma regulation. PLoS ONE.

[43] Bokhari HA (2020). Whole exome sequencing of a Saudi family and systems biology analysis identifies CPED1 as a putative causative gene to celiac disease. Saudi J. Biol. Sci..

[44] Mlecnik B, Galon J, Bindea G (2018). Comprehensive functional analysis of large lists of genes and proteins. J. Proteom..

[45] Palová-Jelínková L (2013). Pepsin digest of wheat gliadin fraction increases production of IL-1β via TLR4/MyD88/TRIF/MAPK/NF-κB signaling pathway and an NLRP3 inflammasome activation. PLoS ONE.

[46] Hoyer KK, Kuswanto WF, Gallo E, Abbas AK (2009). Distinct roles of helper T-cell subsets in a systemic autoimmune disease. Blood.

[47] Abadie V (2020). IL-15, gluten and HLA-DQ8 drive tissue destruction in coeliac disease. Nature.

[48] Barrat FJ, Crow MK, Ivashkiv LB (2019). Interferon target-gene expression and epigenomic signatures in health and disease. Nat. Immunol..

[49] Pascual V (2016). Different gene expression signatures in children and adults with celiac disease. PLoS ONE.

[50] Powell MD, Read KA, Sreekumar BK, Jones DM, Oestreich KJ (2019). IL-12 signaling drives the differentiation and function of a TH1-derived TFH1-like cell population. Sci. Rep..

[51] Uzel G (2013). Dominant gain-of-function STAT1 mutations in FOXP3 wild-type immune dysregulation-polyendocrinopathy-enteropathy-X-linked-like syndrome. J. Allergy Clin. Immunol..

[52] Shalimar DM (2013). Mechanism of villous atrophy in celiac disease: role of apoptosis and epithelial regeneration. Arch. Pathol. Lab. Med..

[53] Monte M (2003). The cell cycle-regulated protein human GTSE-1 controls DNA damage-induced apoptosis by affecting p53 function. J. Biol. Chem..

[54] Xu S (2019). The p53/miRNAs/Ccna2 pathway serves as a novel regulator of cellular senescence: complement of the canonical p53/p21 pathway. Aging Cell.

[55] Zuccotti G (2013). Intakes of nutrients in Italian children with celiac disease and the role of commercially available gluten-free products. J. Hum. Nutr. Dietetics.

[56] Leonard MM (2019). RNA sequencing of intestinal mucosa reveals novel pathways functionally linked to celiac disease pathogenesis. PLoS ONE.

[57] Naik RD, Seidner DL, Adams DW (2018). Nutritional consideration in celiac disease and nonceliac gluten sensitivity. Gastroenterol. Clin. North Am..

[58] Krizkova S (2012). Metallothioneins and zinc in cancer diagnosis and therapy. Drug Metab. Rev..

[59] Cooke AL (2018). A thumbwheel mechanism for APOA1 activation of LCAT activity in HDL. J. Lipid. Res..

[60] Gordon T, Castelli WP, Hjortland MC, Kannel WB, Dawber TR (1977). High density lipoprotein as a protective factor against coronary heart disease. The Framingham Study. Am. J. Med..

[61] Caliskan Z (2019). Lipid profile, atherogenic indices, and their relationship with epicardial fat thickness and carotid intima-media thickness in celiac disease. Northern Clin. Istanbul.

