# Supplementary material for: Exploring celiac disease candidate pathways by global gene expression profiling and gene network cluster analysis
Source: Sci Rep. 2020 Oct 1;10:16290. doi: 10.1038/s41598-020-73288-6 (PMC7529771; doi:10.1038/s41598-020-73288-6)
Supplement: Supplementary file 1 — Supplementary information. [file 41598_2020_73288_MOESM1_ESM.docx]

**Supporting Information, A**

**Exploring Novel Celiac Disease Candidate Pathways by Global Gene Expression Profiling and Network Gene-Cluster analysis**

Babajan Banaganapalli ^#1,2^, Haifa M. Mansour^#1^, Arif Mohammed^3^, Arwa Mastoor Alharthi^2,4^, Nada M. Aljuaid^5^, Khalidah Nasser^2,6^, Aftab Ahmad^7^, Omar Saadah^8^, Jumana Yousuf Al-Aama^1,2^, Ramu Elango^1,2$^ and Noor Ahmad Shaik ^1,2$^

*** Corresponding authors

**Prof. Noor A Shaik**

**Dr. Ramu Elango**

Department of Genetic Medicine,

Faculty of Medicine,

King Abdulaziz University,

Jeddah-21589, Saudi Arabia.

Email: [relango@kau.edu.sa](mailto:relango@kau.edu.sa)

Department of Genetic Medicine,

Faculty of Medicine,

King Abdulaziz University,

Jeddah-21589, Saudi Arabia.

Email: [nshaik@kau.edu.sa](mailto:nshaik@kau.edu.sa)

Table S1: The functional annotations of PPI networks (from upregulated genes) related to cell division.

| **Ontology Source** | **Term ID** | **GO Term** | **Term PValue** | **FDR** | **Nr. Genes** | **Associated Hub Genes** |
| --- | --- | --- | --- | --- | --- | --- |
| Biological processes | GO:0000280 | nuclear division | 1.73 X 10^-13^ | 8.65 X 10^-13^ | 22 | [ASPM, BIRC5, BUB1, CCNA2, CCNB1, CDC20, CDC25B, CENPE, CKS2, DLGAP5, KIF11, KIF4A, MAD2L1, MKI67, NOS2, PBK, PRC1, PTTG1, RACGAP1, SGO2, TOP2A, TTK] |
|  | GO:0140014 | mitotic nuclear division | 2.08 X 10^-10^ | 5.19 X 10^-10^ | 16 | [BIRC5, BUB1, CCNA2, CCNB1, CDC20, CENPE, DLGAP5, KIF11, KIF4A, MAD2L1, MKI67, PBK, PRC1, PTTG1, RACGAP1, TTK] |
|  | GO:0000819 | sister chromatid segregation | 9.39 X 10^-10^ | 1.57 X 10^-9^ | 13 | [BUB1, CCNB1, CDC20, CENPE, DLGAP5, KIF4A, MAD2L1, PRC1, PTTG1, RACGAP1, SGO2, TOP2A, TTK] |
|  | GO:0051983 | regulation of chromosome segregation | 3.75 X 10^-9^ | 4.68 X 10^-9^ | 10 | [BUB1, CCNB1, CDC20, CENPE, DLGAP5, MAD2L1, MKI67, PTTG1, RACGAP1, TTK] |
|  | GO:0051783 | regulation of nuclear division | 5.62 X 10^-9^ | 2.81 X 10^-8^ | 13 | [BUB1, CCNA2, CCNB1, CDC20, CENPE, DLGAP5, KIF11, MAD2L1, MKI67, NOS2, PBK, PTTG1, TTK] |
| Molecular Function | GO:0052548 | regulation of endopeptidase activity | 4.46 X 10^-5^ | 1.11 x 10^-4^ | 12 | [BIRC3, BIRC5, CARD16, CASP1, CCNA2, JAK2, PMAIP1, PSMB9, PTTG1, SPINK4, TAP1, TFRC] |
|  | GO:2000116 | regulation of cysteine-type endopeptidase activity | 7.00 X 10^-5^ | 1.17 x 10^-4^ | 9 | [BIRC3, CARD16, CASP1, CCNA2, JAK2, PMAIP1, PSMB9, TAP1, TFRC] |
|  | GO:0052547 | regulation of peptidase activity | 7.60 X 10^-5^ | 9.50 X 10^-5^ | 12 | [BIRC3, BIRC5, CARD16, CASP1, CCNA2, JAK2, PMAIP1, PSMB9, PTTG1, SPINK4, TAP1, TFRC] |
|  | GO:0043281 | regulation of cysteine-type endopeptidase activity involved in apoptotic process | 2.05 x 10^-4^ | 2.56 x 10^-4^ | 8 | [BIRC3, CARD16, CASP1, CCNA2, JAK2, PMAIP1, TAP1, TFRC] |
|  | GO:0097153 | cysteine-type endopeptidase activity involved in apoptotic process | 2.46 x 10^-4^ | 3.08 x 10^-4^ | 8 | [BIRC3, CARD16, CASP1, CCNA2, JAK2, PMAIP1, TAP1, TFRC] |
| Cellular Component | GO:0005819 | spindle | 5.63 X 10^-7^ | 2.81 X 10^-6^ | 13 | [ASPM, BIRC5, CCNB1, CDC20, CDC25B, CENPE, DLGAP5, KIF11, KIF4A, MAD2L1, PRC1, RACGAP1, TTK] |
|  | GO:0030496 | midbody | 3.05 X 10^-6^ | 7.63 X 10^-6^ | 9 | [ASPM, BIRC3, BIRC5, CENPE, CEP55, KIF4A, LAP3, PRC1, RACGAP1] |
|  | GO:0000777 | condensed chromosome kinetochore | 3.83 X 10^-6^ | 6.39 X 10^-6^ | 7 | [BIRC5, BUB1, CCNB1, CENPE, CENPW, MAD2L1, SGO2] |
|  | GO:0000776 | kinetochore | 3.99 X 10^-6^ | 9.97 X 10^-6^ | 8 | [BIRC5, BUB1, CCNB1, CENPE, CENPW, MAD2L1, SGO2, TTK] |
|  | GO:0000779 | condensed chromosome, centromeric region | 1.04 X 10^-5^ | 1.74 X 10^-5^ | 7 | [BIRC5, BUB1, CCNB1, CENPE, CENPW, MAD2L1, SGO2] |
| KEGG pathways | KEGG:04110 | Cell cycle | 6.60 X 10^-7^ | 3.30 X 10^-6^ | 9 | [BUB1, CCNA2, CCNB1, CDC20, CDC25B, E2F5, MAD2L1, PTTG1, TTK] |
|  | KEGG:04914 | Progesterone-mediated oocyte maturation | 1.28 x 10^-3^ | 6.41 x 10^-3^ | 5 | [BUB1, CCNA2, CCNB1, CDC25B, MAD2L1] |
|  | KEGG:04115 | p53 signaling pathway | 2.84 x 10^-3^ | 7.11 x 10^-3^ | 4 | [CCNB1, GTSE1, PMAIP1, RRM2] |
|  | KEGG:04114 | Oocyte meiosis | 3.5.7 x 10-3 | 5.95 x 10^-3^ | 5 | [BUB1, CCNB1, CDC20, MAD2L1, PTTG1] |
|  | KEGG:04210 | Apoptosis | 2.55 x 10^-2^ | 2.55 x 10^-2^ | 4 | [BIRC3, BIRC5, GZMB, PMAIP1] |

Table S2: The functional annotations of PPI networks (from upregulated genes) related to immune system.

| **Ontology Source** | **Term ID** | **GO Term** | **Term PValue** | **FDR** | **Gene Count** | **Associated Hub Genes** |
| --- | --- | --- | --- | --- | --- | --- |
| Biological Processes | GO:0034341 | response to interferon-gamma | 8.90 X 10^-11^ | 5.34 X 10+ | 14 | [CASP1, CCL5, GBP1, GBP2, GBP4, IFITM1, IFNG, IRF1, IRF8, JAK2, NLRC5, NOS2, OAS2, STAT1] |
|  | GO:0071346 | cellular response to interferon-gamma | 3.30 X 10^-10^ | 9.90 X 10^-10^ | 13 | [CASP1, CCL5, GBP1, GBP2, GBP4, IFNG, IRF1, IRF8, JAK2, NLRC5, NOS2, OAS2, STAT1] |
|  | GO:0071357 | cellular response to type I interferon | 7.05 X 10^-9^ | 1.41 X 10^-8^ | 9 | [EGR1, GBP2, IFITM1, IRF1, IRF8, MMP12, NLRC5, OAS2, STAT1] |
|  | GO:0060337 | type I interferon signaling pathway | 7.05 X 10^-9^ | 1.41 X 10^-8^ | 9 | [EGR1, GBP2, IFITM1, IRF1, IRF8, MMP12, NLRC5, OAS2, STAT1] |
|  | GO:0034340 | response to type I interferon | 1.06 X 10^-8^ | 1.59 X 10^-8^ | 9 | [EGR1, GBP2, IFITM1, IRF1, IRF8, MMP12, NLRC5, OAS2, STAT1] |
|  | GO:0060333 | interferon-gamma-mediated signaling pathway | 1.15 X 10^-8^ | 1.38 X 10^-8^ | 9 | [GBP1, GBP2, IFNG, IRF1, IRF8, JAK2, NLRC5, OAS2, STAT1] |
|  | GO:0001959 | regulation of cytokin x 10-mediated signaling pathway | 7.54 X 10^-8^ | 4.52 X 10^-7^ | 11 | [BIRC3, CARD16, CASP1, CCL5, CD24, IFNG, JAK2, MMP12, NLRC5, PBK, STAT1] |
|  | GO:0032651 | regulation of interleukin-1 beta production | 3.64 X 10^-6^ | 1.09 X 10^-5^ | 7 | [CARD16, CASP1, EGR1, JAK2, LPL, PBK, TFRC] |
|  | GO:0032611 | interleukin-1 beta production | 7.35 X 10^-6^ | 1.47 X 10^-5^ | 7 | [CARD16, CASP1, EGR1, JAK2, LPL, PBK, TFRC] |
| Molecular Functions | GO:0042379 | chemokine receptor binding | 1.14 X 10^-5^ | 1.70 X 10^-5^ | 6 | [ACKR4, CCL5, CXCL10, CXCL11, CXCL9, STAT1] |
|  | GO:0001664 | G protein-coupled receptor binding | 3.33 X 10^-5^ | 4.00 X 10^-5^ | 10 | [ACKR4, ASPM, BIRC3, CCL5, CLIC6, CXCL10, CXCL11, CXCL9, JAK2, STAT1] |
|  | GO:0005126 | cytokine receptor binding | 3.74 X 10^-5^ | 1.12 x 10-4 | 10 | [ACKR4, BIRC3, CCL5, CXCL10, CXCL11, CXCL9, IFNG, JAK2, STAT1, TNFSF13B] |
|  | GO:0008009 | chemokine activity | 4.64 x 10^-4^ | 1.04 x 10‑ | 4 | [CCL5, CXCL10, CXCL11, CXCL9] |
|  | GO:0005125 | cytokine activity | 9.42 x 10^-4^ | 1.41 x 10^-3^ | 7 | [CCL5, CXCL10, CXCL11, CXCL9, IFNG, NAMPT, TNFSF13B] |
| KEGG pathways | KEGG:04621 | NOD-like receptor signaling pathway | 1.60 X 10^-6^ | 7.22 X 10^-6^ | 10 | [BIRC3, CARD16, CASP1, CCL5, GBP1, GBP2, GBP4, NAMPT, OAS2, STAT1] |
|  | KEGG:04658 | Th1 and Th2 cell differentiation | 9.22 x 10^-4^ | 1.65 x 10^-3^ | 5 | [CD3D, CD3G, IFNG, JAK2, STAT1] |
|  | KEGG:04657 | IL-17 signaling pathway | 9.68 x 10^-4^ | 1.24 x 10^-3^ | 5 | [CXCL10, IFNG, LCN2, MMP1, MMP3] |
|  | KEGG:04620 | Toll-like receptor signaling pathway | 1.59 x 10^-3^ | 1.79 x 10^-3^ | 5 | [CCL5, CXCL10, CXCL11, CXCL9, STAT1] |
|  | KEGG:04659 | Th17 cell differentiation | 1.81 x 10^-3^ | 3.10 x 10^-3^ | 5 | [CD3D, CD3G, IFNG, JAK2, STAT1] |
| Immune System Processes | GO:0034341 | response to interferon-gamma | 1.16 X 10^-6^ | 1.39 X 10^-5^ | 14 | [CASP1, CCL5, GBP1, GBP2, GBP4, IFITM1, IFNG, IRF1, IRF8, JAK2, NLRC5, NOS2, OAS2, STAT1] |
|  | GO:0060337 | type I interferon signaling pathway | 4.92 X 10^-6^ | 2.95 X 10^-5^ | 9 | [EGR1, GBP2, IFITM1, IRF1, IRF8, MMP12, NLRC5, OAS2, STAT1] |
|  | GO:0034340 | response to type I interferon | 7.22 X 10^-6^ | 2.89 X 10^-5^ | 9 | [EGR1, GBP2, IFITM1, IRF1, IRF8, MMP12, NLRC5, OAS2, STAT1] |
|  | GO:0060333 | interferon-gamma-mediated signaling pathway | 7.78 X 10^-6^ | 2.33 X 10^-5^ | 9 | [GBP1, GBP2, IFNG, IRF1, IRF8, JAK2, NLRC5, OAS2, STAT1] |
|  | GO:0060330 | regulation of response to interferon-gamma | 8.77 x 10^-4^ | 1.75 x 10-^3^ | 4 | [IFNG, JAK2, NLRC5, STAT1] |
|  | GO:0060334 | regulation of interferon-gamma-mediated signaling pathway | 8.77 x 10^-4^ | 1.75 x 10^-3^ | 4 | [IFNG, JAK2, NLRC5, STAT1] |
|  | GO:0042102 | positive regulation of T cell proliferation | 2.27 x 10^-2^ | 3.03 x 10^-2^ | 5 | [CCL5, CD24, CD274, TFRC, TNFSF13B] |
|  | GO:0002478 | antigen processing and presentation of exogenous peptide antigen | 5.12 x 10^-2^ | 6.14 x 10^-2^ | 6 | [CENPE, KIF11, KIF4A, PSMB9, RACGAP1, TAP1] |
|  | GO:0042129 | regulation of T cell proliferation | 5.61 x 10^-2^ | 6.12 x 10^-2^ | 6 | [CCL5, CD24, CD274, IRF1, TFRC, TNFSF13B] |
| Human diseases | C1868139 | Medullary cystic kidney disease 1 | 6.89 x 10^-3^ | 1.37 x 10^-2^ | 1 | [MUC1] |
|  | C3280913 | Coronary heart disease 6 | 6.89 x 10^-3^ | 1.37 x 10^-2^ | 1 | [MMP3] |
|  | C3810147 | Immunodeficiency 19 | 6.89 x 10^-3^ | 1.37 x 10^-2^ | 1 | [CD3D] |
|  | C1837065 | Cd8 deficiency, familial | 6.89 x 10^-3^ | 1.37 x 10^-2^ | 1 | [CD8A] |
|  | C2677770 | Asthma-related traits, susceptibility to, 7 | 6.89 x 10^-3^ | 1.37 x 10^-2^ | 1 | [CHI3L1] |

Table S3: The functional annotations of PPI networks (from downregulated genes) related to metabolism and absorption.

| **Ontology Source** | **Term ID** | **GO Term** | **Term PValue** | **FDR** | **Nr. Genes** | **Associated Hub Genes** |
| --- | --- | --- | --- | --- | --- | --- |
| Biological Processes | GO:0044242 | cellular lipid catabolic process | 1.93 X 10^-13^ | 1.16 X 10^-12^ | 22 | [ABHD2, ABHD6, ACADM, ACAT1, ACOX1, APOA1, APOA4, APOB, APOC3, ASAH2B, CYP4F2, ENPP2, ENPP7, GC, GDPD1, GPCPD1, NT5E, PCK1, PHYH, PLA2G4C, SCARB1, SOAT2] |
|  | GO:0032787 | monocarboxylic acid metabolic process | 5.31 X 10^-13^ | 1.59 X 10^-12^ | 35 | [ABHD2, ACADM, ACAT1, ACOX1, ACSF2, ACSL1, ADH6, ALDH1A3, ALDOB, APOA4, APOC3, ASAH2B, CD36, CYP1A1, CYP2C9, CYP2J2, CYP3A4, CYP4F2, DGAT2, GPX4, NAAA, PCK1, PDK4, PFKFB4, PHYH, PLA2G4C, PON3, RBP1, RGN, SOAT2, SORD, SULT2A1, UGT2A3, UGT2B28, VNN1] |
|  | GO:0010273 | detoxification of copper ion | 4.12 X 10^-12^ | 8.24 X 10^-12^ | 8 | [MT1E, MT1F, MT1G, MT1H, MT1HL1, MT1M, MT1X, MT2A] |
|  | GO:0098754 | detoxification | 4.21 X 10^-12^ | 6.32 X 10^-12^ | 17 | [ADH4, APOA4, CD36, GPX3, GPX4, GSTT1, MT1E, MT1F, MT1G, MT1H, MT1HL1, MT1M, MT1X, MT2A, PON3, PRXL2A, RGN] |
|  | GO:0061687 | detoxification of inorganic compound | 7.05 X 10^-12^ | 8.47 X 10^-12^ | 8 | [MT1E, MT1F, MT1G, MT1H, MT1HL1, MT1M, MT1X, MT2A] |
| Molecular Functions | GO:0015294 | solute:cation symporter activity | 1.16 X 10^-7^ | 3.49 X 10^-7^ | 11 | [SLC10A2, SLC13A2, SLC15A1, SLC17A4, SLC23A1, SLC28A2, SLC2A12, SLC4A4, SLC4A7, SLC5A9, SLC6A4] |
|  | GO:0015293 | symporter activity | 3.94 X 10^-7^ | 7.89 X 10^-7^ | 12 | [SLC10A2, SLC13A2, SLC15A1, SLC17A4, SLC22A4, SLC23A1, SLC28A2, SLC2A12, SLC4A4, SLC4A7, SLC5A9, SLC6A4] |
|  | GO:0015370 | solute:sodium symporter activity | 4.72 X 10^-7^ | 7.08 X 10^-7^ | 9 | [SLC10A2, SLC13A2, SLC17A4, SLC23A1, SLC28A2, SLC4A4, SLC4A7, SLC5A9, SLC6A4] |
|  | GO:0043178 | alcohol binding | 2.55 X 10^-6^ | 3.07 X 10^-6^ | 9 | [ACAT1, ADH4, APOA1, APOA4, APOC3, C8G, GRAMD1B, RBP1, SOAT2] |
|  | GO:0015291 | secondary active transmembrane transporter activity | 3.20 X 10^-6^ | 3.84 X 10^-6^ | 14 | [G6PC, SLC10A2, SLC13A2, SLC15A1, SLC17A4, SLC19A1, SLC22A4, SLC23A1, SLC28A2, SLC2A12, SLC4A4, SLC4A7, SLC5A9, SLC6A4] |
| Cellular Components | GO:0016324 | apical plasma membrane | 2.49 X 10^-12^ | 1.50 X 10^-11^ | 24 | [ABCC2, CD36, CDHR2, CDHR5, CYP4F2, DPEP1, ENPEP, ENPP3, GPX4, MGAM, NAALADL1, PDZK1, SCNN1A, SLC10A2, SLC17A4, SLC19A1, SLC22A4, SLC23A1, SLC2A5, SLC46A1, SLC4A4, SLC4A7, SLC52A3, TRPM6] |
|  | GO:0045177 | apical part of cell | 1.84 X 10^-11^ | 1.11 X 10^-10^ | 25 | [ABCC2, CA2, CD36, CDHR2, CDHR5, CYP4F2, DPEP1, ENPEP, ENPP3, GPX4, MGAM, NAALADL1, PDZK1, SCNN1A, SLC10A2, SLC17A4, SLC19A1, SLC22A4, SLC23A1, SLC2A5, SLC46A1, SLC4A4, SLC4A7, SLC52A3, TRPM6] |
|  | GO:0098862 | cluster of actin-based cell projections | 1.01 X 10^-7^ | 3.05 X 10^-7^ | 13 | [CD36, CDHR2, CDHR5, CYBRD1, ENPEP, MME, PDZK1, SLC15A1, SLC46A1, SLC4A4, SLC4A7, SOAT2, TRPM6] |
|  | GO:0005903 | brush border | 1.13 X 10^-7^ | 2.27 X 10^-7^ | 11 | [CD36, CDHR2, CDHR5, CYBRD1, ENPEP, MME, PDZK1, SLC15A1, SLC46A1, SOAT2, TRPM6] |
|  | GO:0031526 | brush border membrane | 4.69 X 10^-6^ | 7.04 X 10^-6^ | 7 | [CD36, CDHR2, CDHR5, CYBRD1, PDZK1, SLC46A1, TRPM6] |
|  | GO:0005902 | microvillus | 1.61 X 10^-5^ | 1.94 X 10^-5^ | 8 | [CA2, CDHR2, CDHR5, DPEP1, ENPP7, PDZK1, SCARB1, SLC10A2] |
|  | GO:0031528 | microvillus membrane | 2.96 X 10-5 | 3.56 X 10^-5^ | 5 | [CDHR2, CDHR5, DPEP1, PDZK1, SCARB1] |
| KEGG pathways | KEGG:04978 | Mineral absorption | 2.29 X 10^-10^ | 1.84 X 10^-9^ | 11 | [CYBRD1, MT1E, MT1F, MT1G, MT1H, MT1HL1, MT1M, MT1X, MT2A, SLC46A1, TRPM6] |
|  | KEGG:00980 | Metabolism of xenobiotics by cytochrome P450 | 1.54 X 10^-9^ | 6.18 X 10^-9^ | 12 | [ADH4, ADH6, ALDH1A3, CYP1A1, CYP2C9, CYP3A4, DHDH, EPHX1, GSTT1, SULT2A1, UGT2A3, UGT2B28] |
|  | KEGG:04977 | Vitamin digestion and absorption | 1.62 X 10^-9^ | 4.34 X 10-9 | 8 | [APOA1, APOA4, APOB, SCARB1, SLC19A1, SLC23A1, SLC46A1, SLC52A3] |
|  | KEGG:05204 | Chemical carcinogenesis | 3.81 X 10^-9^ | 7.64 X 10^-9^ | 12 | [ADH4, ADH6, ALDH1A3, CYP1A1, CYP2C9, CYP3A4, CYP3A7-CYP3A51P, EPHX1, GSTT1, SULT2A1, UGT2A3, UGT2B28] |
|  | KEGG:00982 | Drug metabolism | 1.10 X 10^-8^ | 1.77 X 10^-8^ | 11 | [ADH4, ADH6, ALDH1A3, CYP2C9, CYP3A4, FMO4, FMO5, GSTT1, MAOB, UGT2A3, UGT2B28] |
|  | KEGG:04976 | Bile secretion | 1.35 X 10^-7^ | 1.81 X 10^-7^ | 10 | [ABCC2, ABCG2, ADCY9, CA2, CYP3A4, EPHX1, SCARB1, SLC10A2, SLC4A4, SULT2A1] |
|  | KEGG:04975 | Fat digestion and absorption | 1.69 X 10^-7^ | 1.94 X 10^-7^ | 8 | [APOA1, APOA4, APOB, CD36, DGAT2, PLPP1, PLPP3, SCARB1] |
| Human Diseases | C0026705 | Mucopolysaccharidosis, MPS-II | 1.32 x 10^-2^ | 2.65 x 10^-2^ | 1 | [IDS] |
|  | C1836602 | Bruck syndrome 2 | 1.32 x 10^-2^ | 2.65 x 10^-2^ | 1 | [PLOD2] |
|  | C1836916 | Posterior column ataxia with retinitis pigmentosa | 1.32 x 10^-2^ | 2.65 x 10^-2^ | 1 | [FLVCR1] |
|  | C0016751 | Hereditary fructosuria | 1.32 x 10^-2^ | 2.65 x 10^-2^ | 1 | [ALDOB] |
|  | C1842937 | Aural atresia, congenital | 1.32 x 10^-2^ | 2.65 x 10^-2^ | 1 | [TSHZ1] |
|  | C1849678 | Pseudoneonatal adrenoleukodystrophy | 1.32 x 10^-2^ | 2.65 x 10^-2^ | 1 | [ACOX1] |
|  | C1849814 | Phosphoenolpyruvate carboxykinase deficiency, cytosolic | 1.32 x 10^-2^ | 2.65 x 10^-2^ | 1 | [PCK1] |
|  | C1853096 | High density lipoprotein cholesterol level quantitative trait locus 6 | 1.32 x 10^-2^ | 2.65 x 10^-2^ | 1 | [SCARB1] |
|  | C1855229 | Spondylometaphyseal dysplasia Sedaghatian type | 1.32 x 10^-2^ | 2.65 x 10^-2^ | 1 | [GPX4] |
|  | C1857342 | Deafness and myopia | 1.32 x 10^-2^ | 2.65 x 10^-2^ | 1 | [SLITRK6] |
|  | C1859372 | Calcification of joints and arteries | 1.32 x 10^-2^ | 2.65 x 10^-2^ | 1 | [NT5E] |
|  | C1865974 | Hypomagnesemia 1, intestinal | 1.32 x 10^-2^ | 2.65 x 10^-2^ | 1 | [TRPM6] |


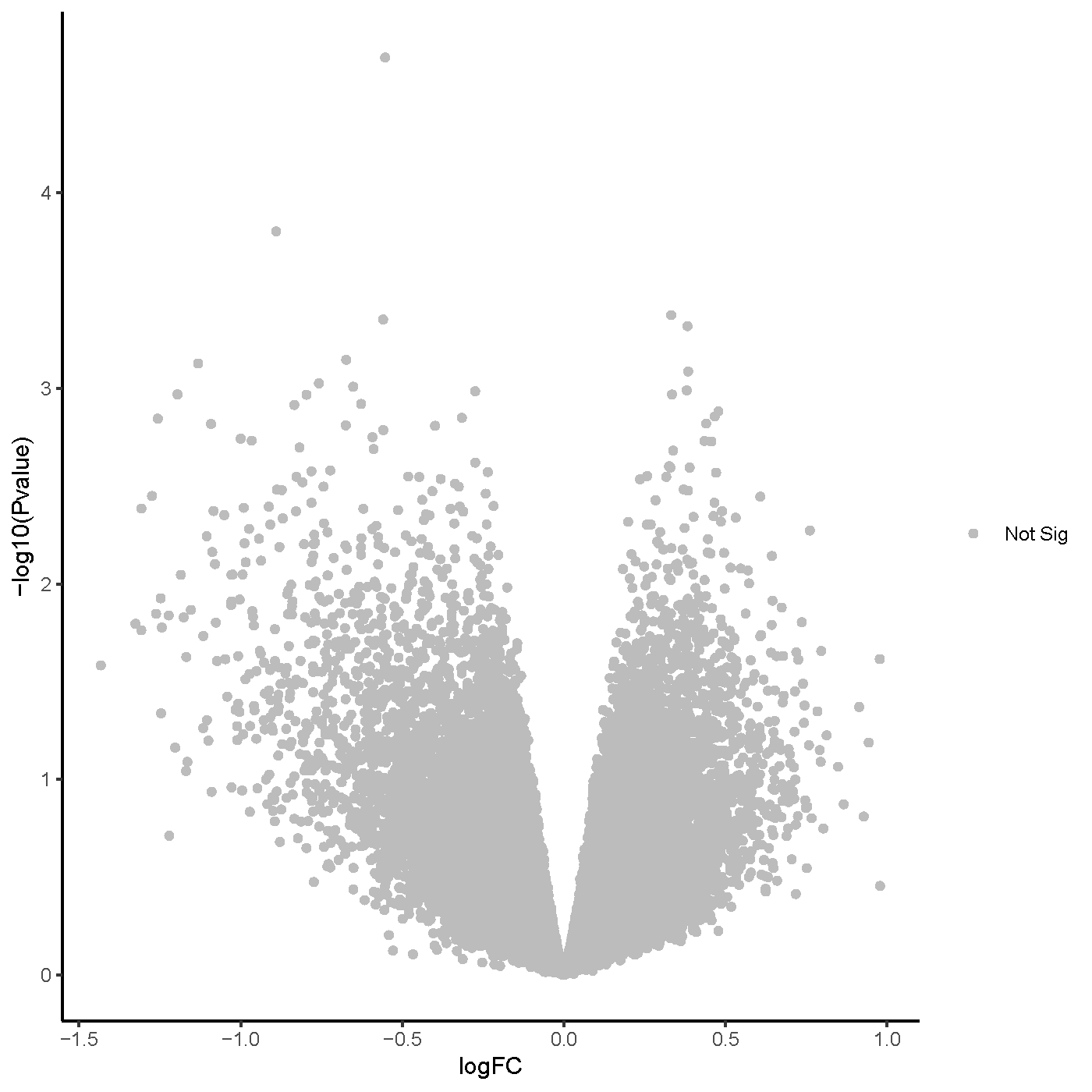


**Figure S1: Volcano plot of invitro gliadin Challenge vs CD Diagnosis**


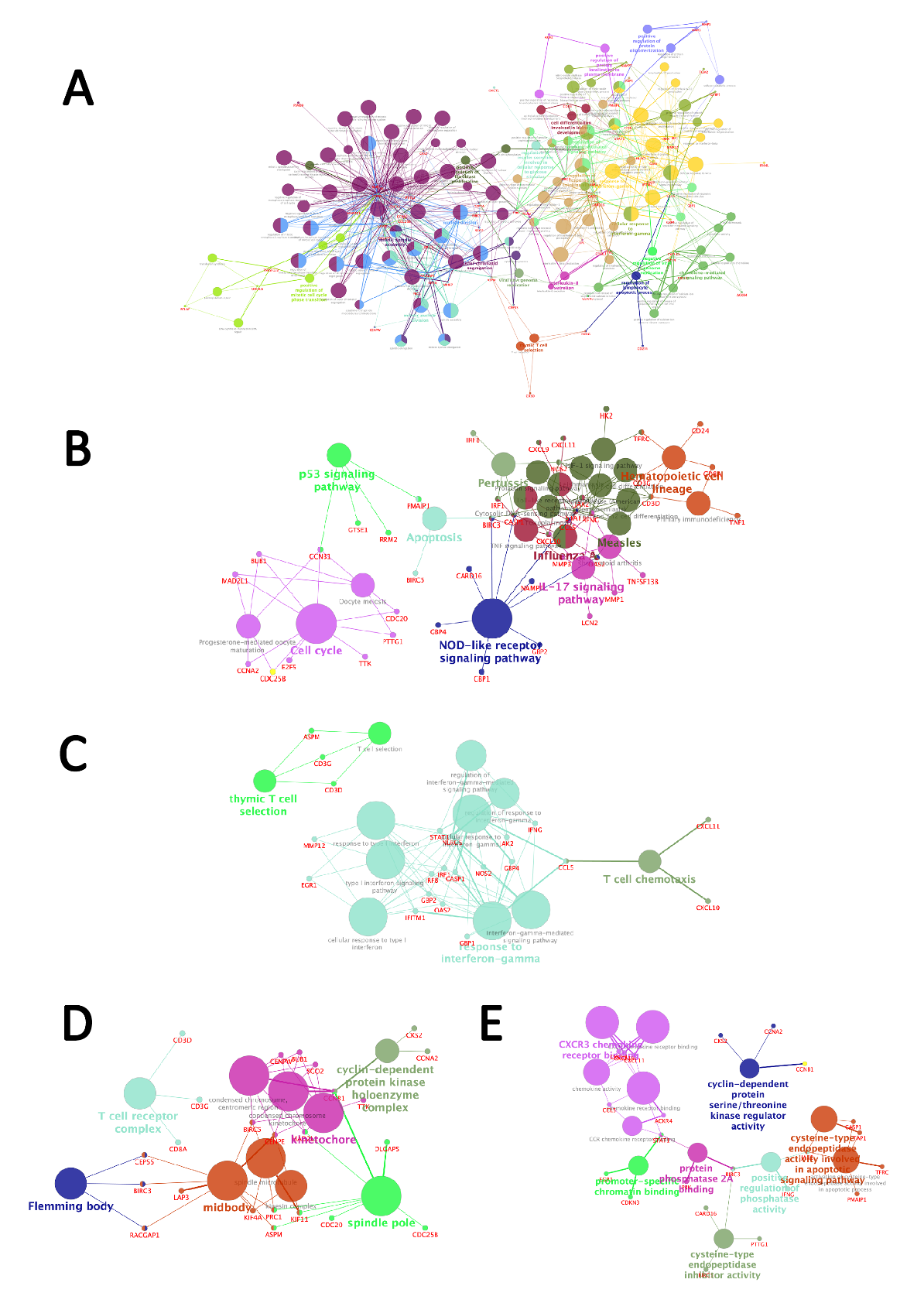


**Figure S2: Functional annotation of gene network interactions from upregulated PPI.**

A) Biological processes. B) KEGG pathways. C) Immune system processes. D) Cell components. E) Molecular functions. (A-E Figures generated using https://cytoscape.org/)


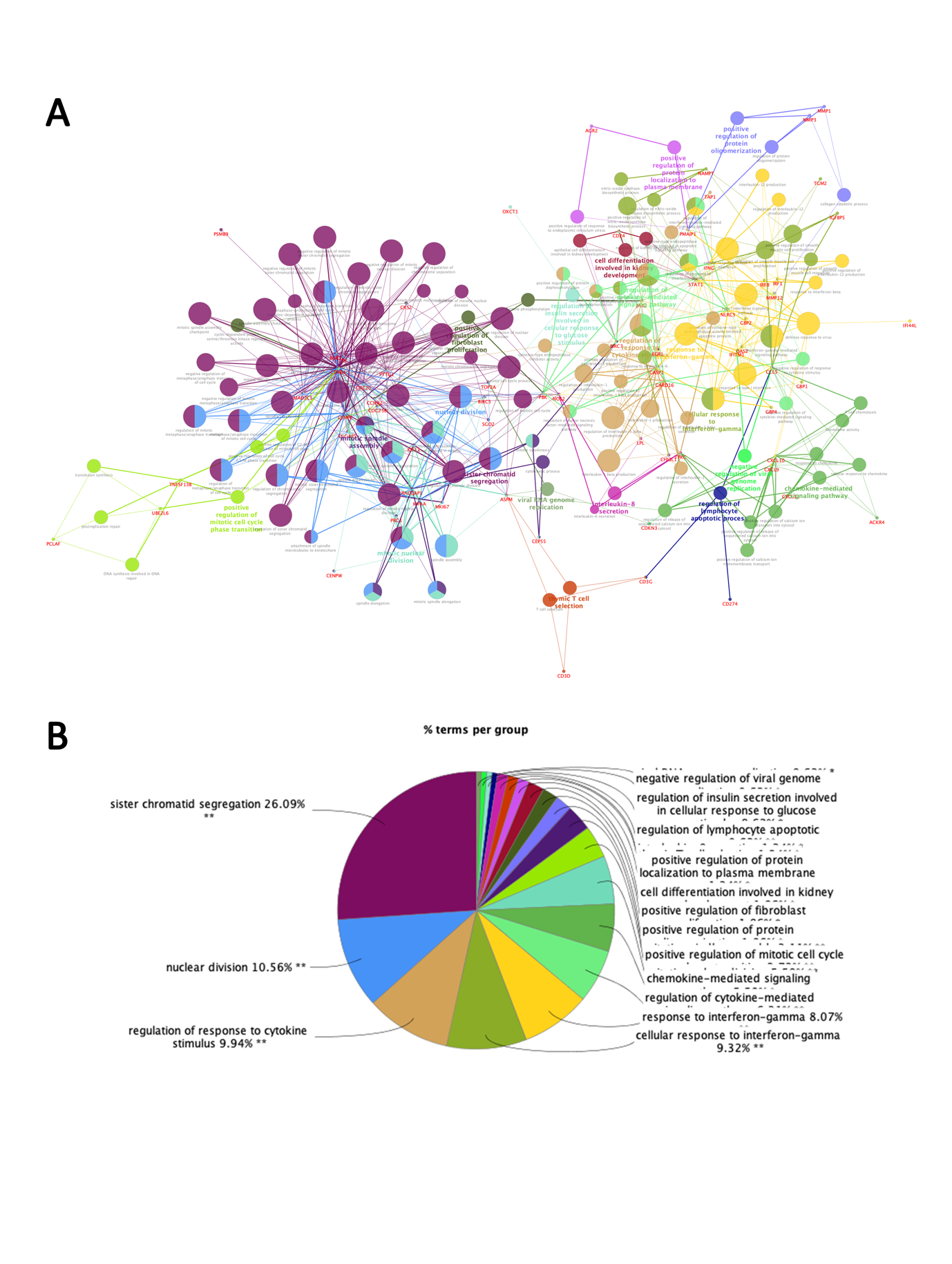


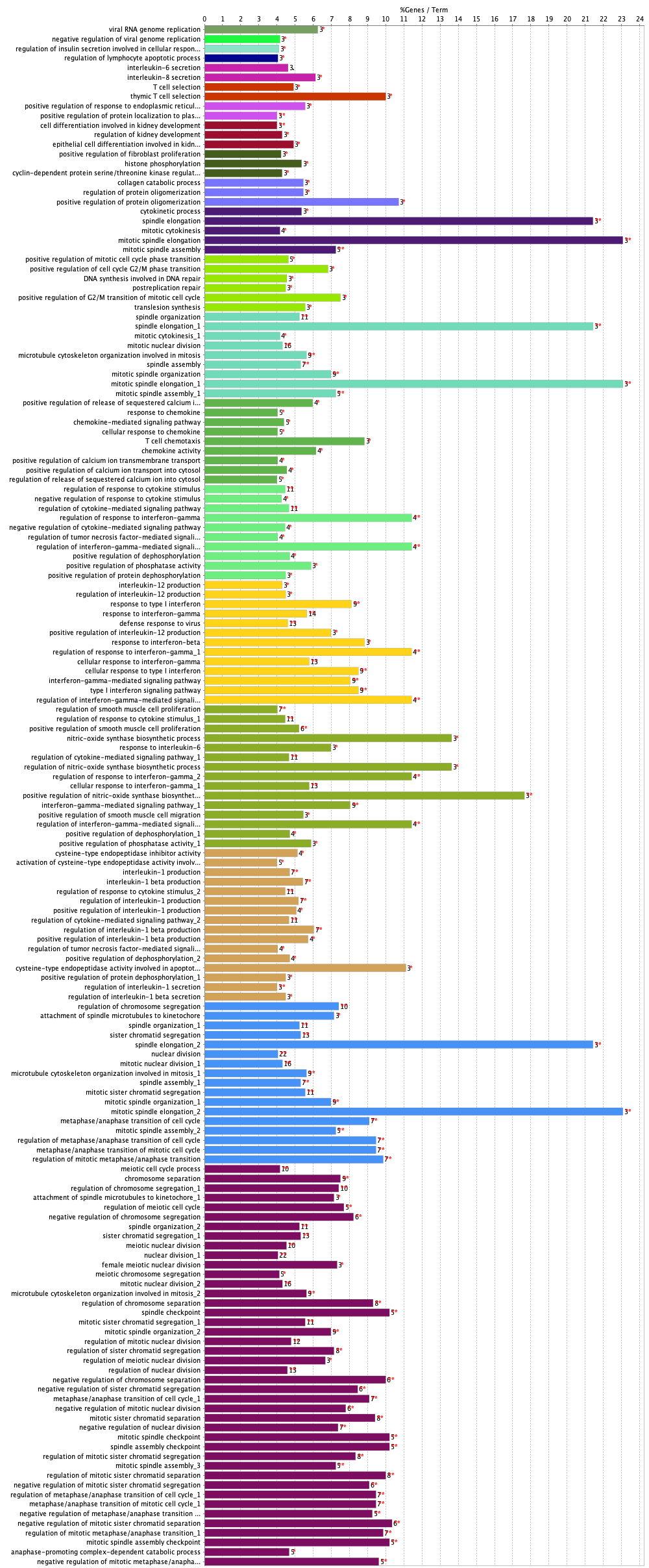


**C**

**Figure S3: Biological processes identified from upregulated PPI.**

- 1. Gene network interactions. B& C) Genes involvement in biological processes in percentage and count. (A-C Figures generated using https://cytoscape.org/)


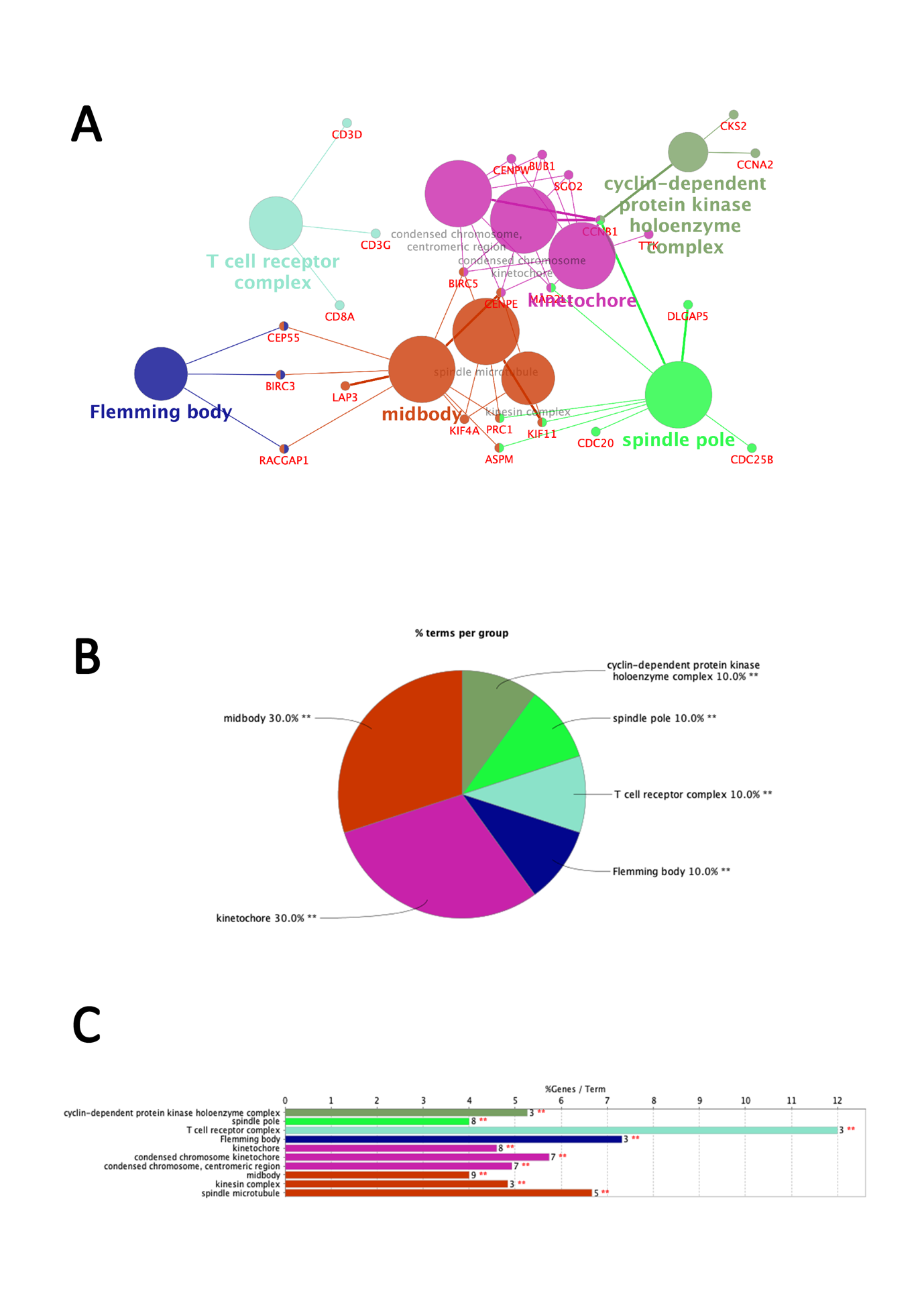
 **Figure S4: Cellular components identified from upregulated PPIs.**

A) Gene network interactions. B& C) Genes involvement in cellular components in percentage and count. (A-C Figures generated using https://cytoscape.org/)


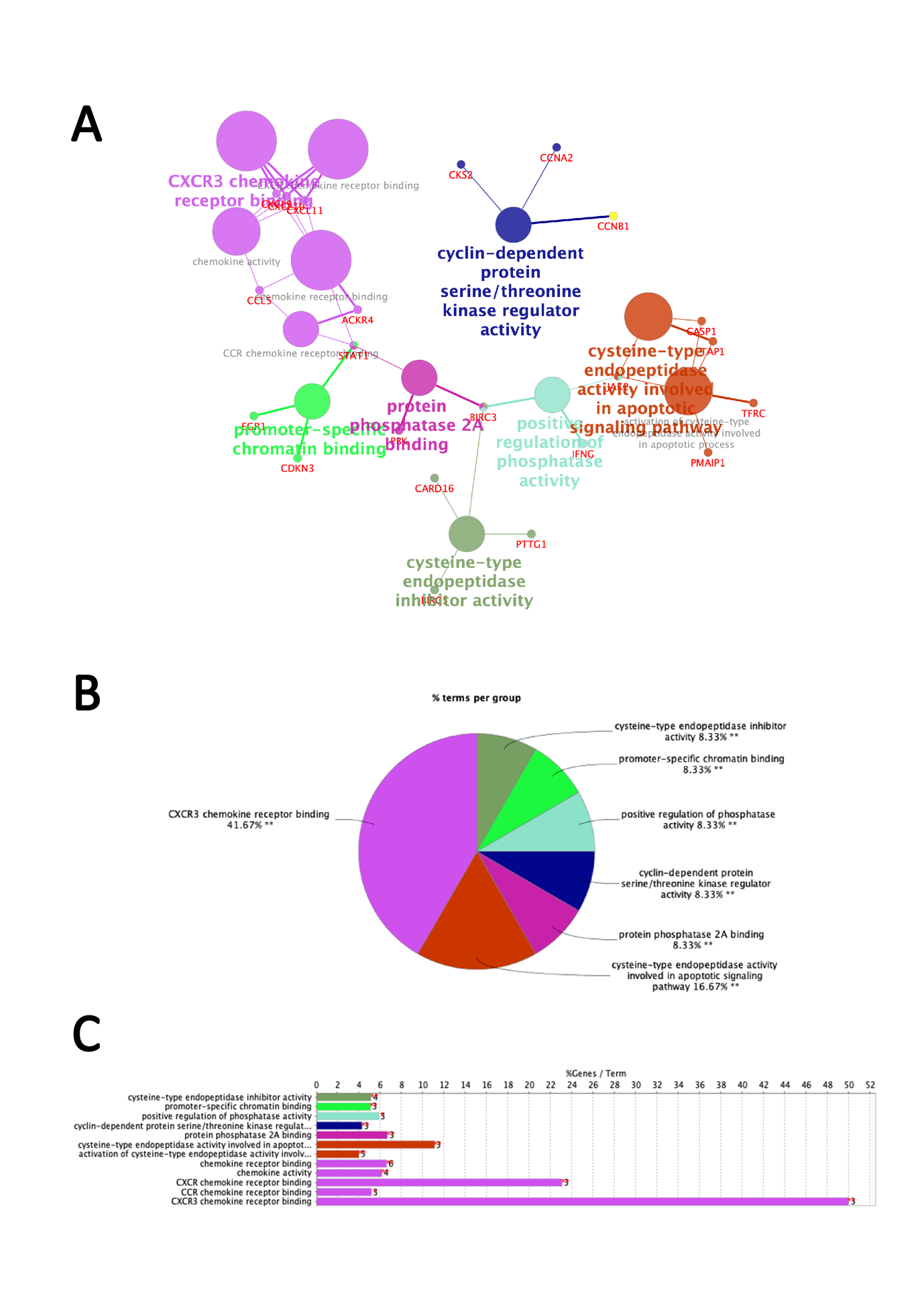
 **Figure S5: Molecular functions identified from upregulated PPI.**

A) Gene network interactions. B& C) Genes involvement in molecular functions in percentage and count. (A-C Figures generated using https://cytoscape.org/)


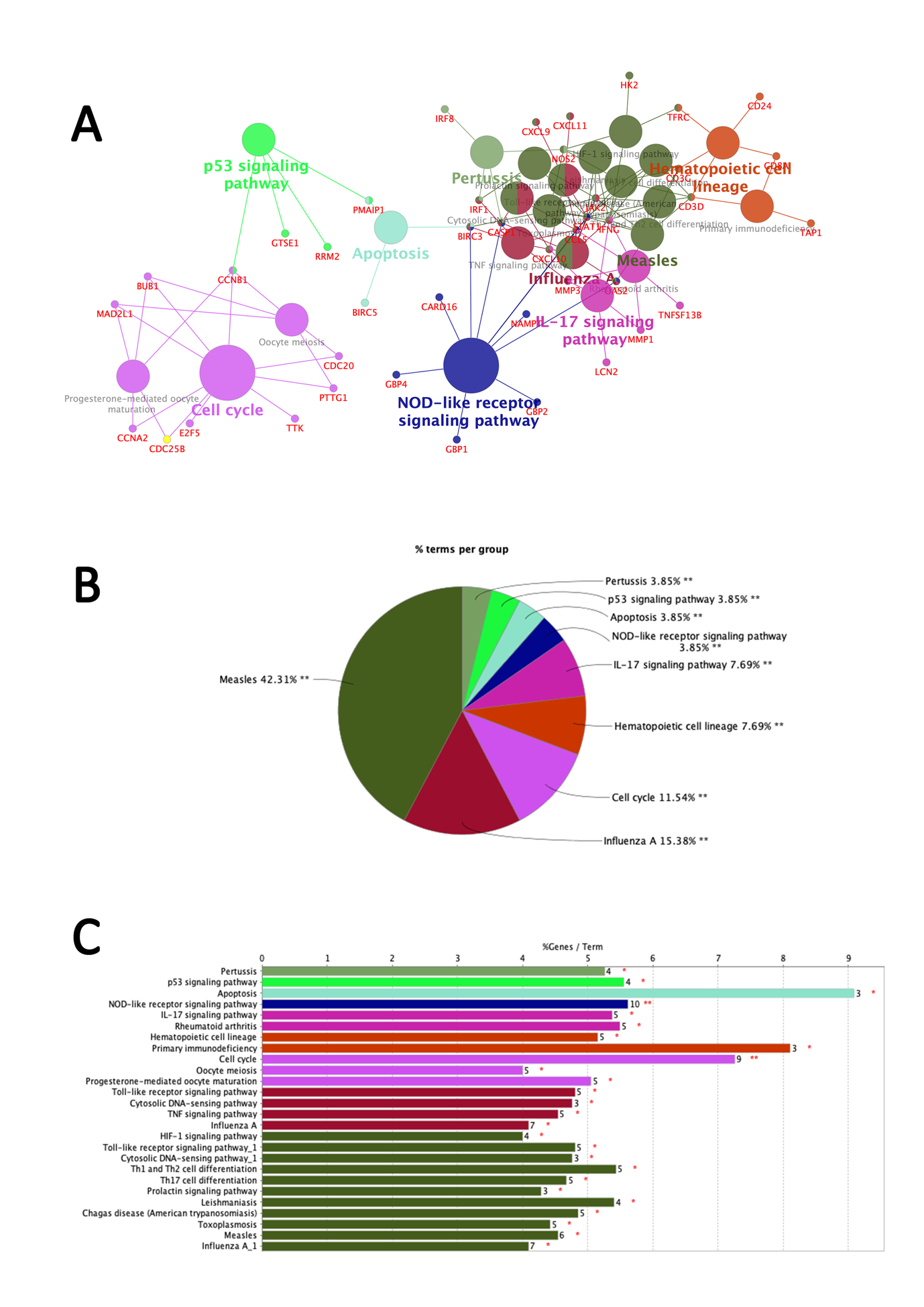


**Figure S6: KEGG pathways identified from upregulated PPI.**

A) Gene network interactions. B& C) Genes involvement in KEGG pathways in percentage and count. (A-C Figures generated using https://cytoscape.org/)


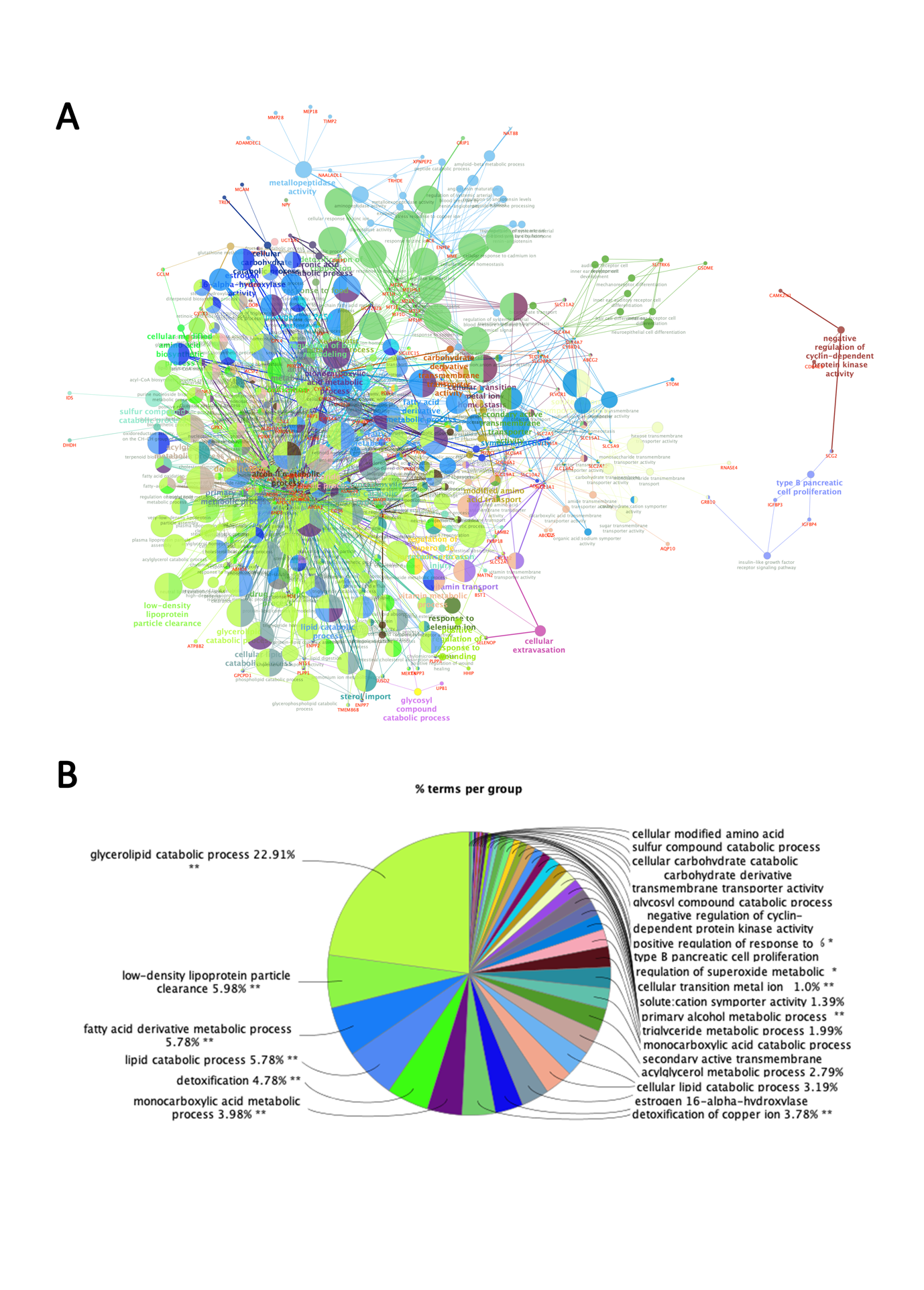


**C**


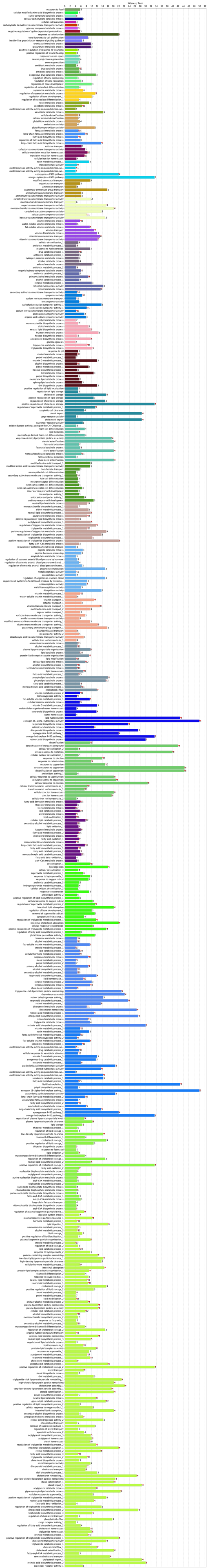

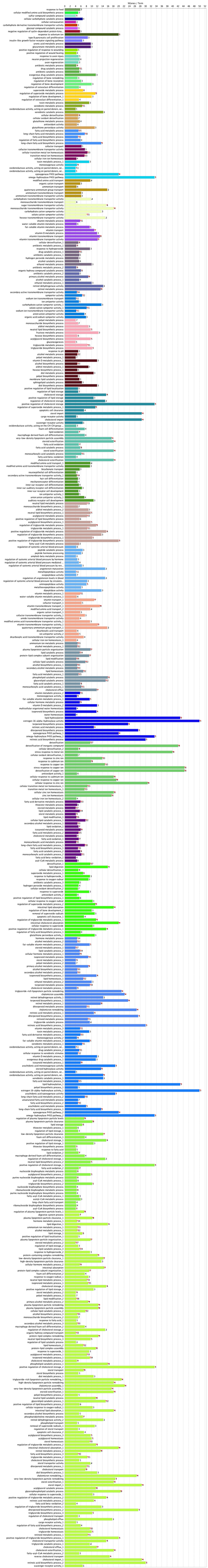


**Figure S7: Biological processes identified from downregulated PPI.**

A) Gene network interactions. B& C) Genes involvement in biological processes in percentage and count. (A-C Figures generated using https://cytoscape.org/)


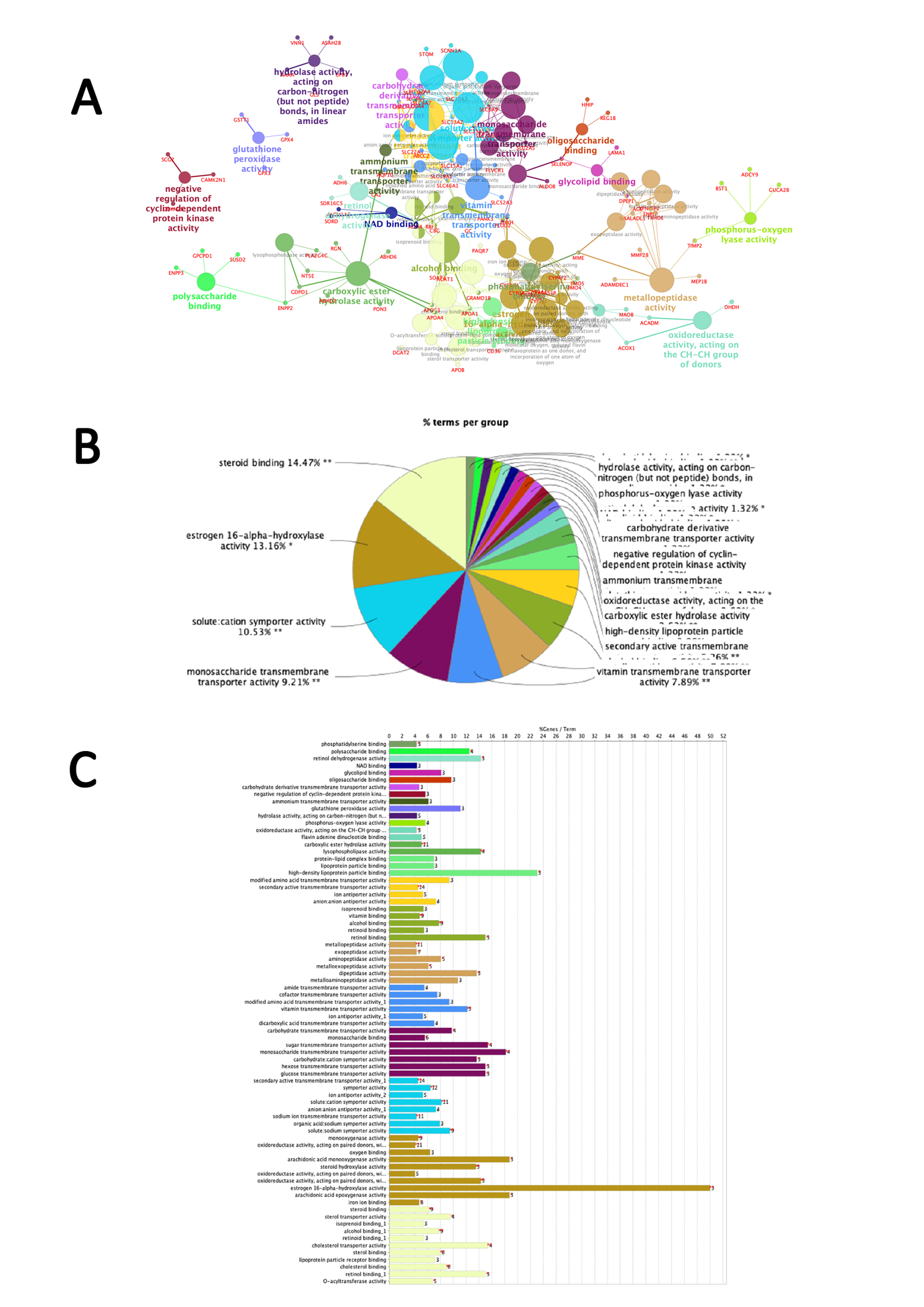


**Figure S8: Molecular functions identified from downregulated PPI.**

A) Gene network interactions. B& C) Genes involvement in molecular functions in percentage and count.


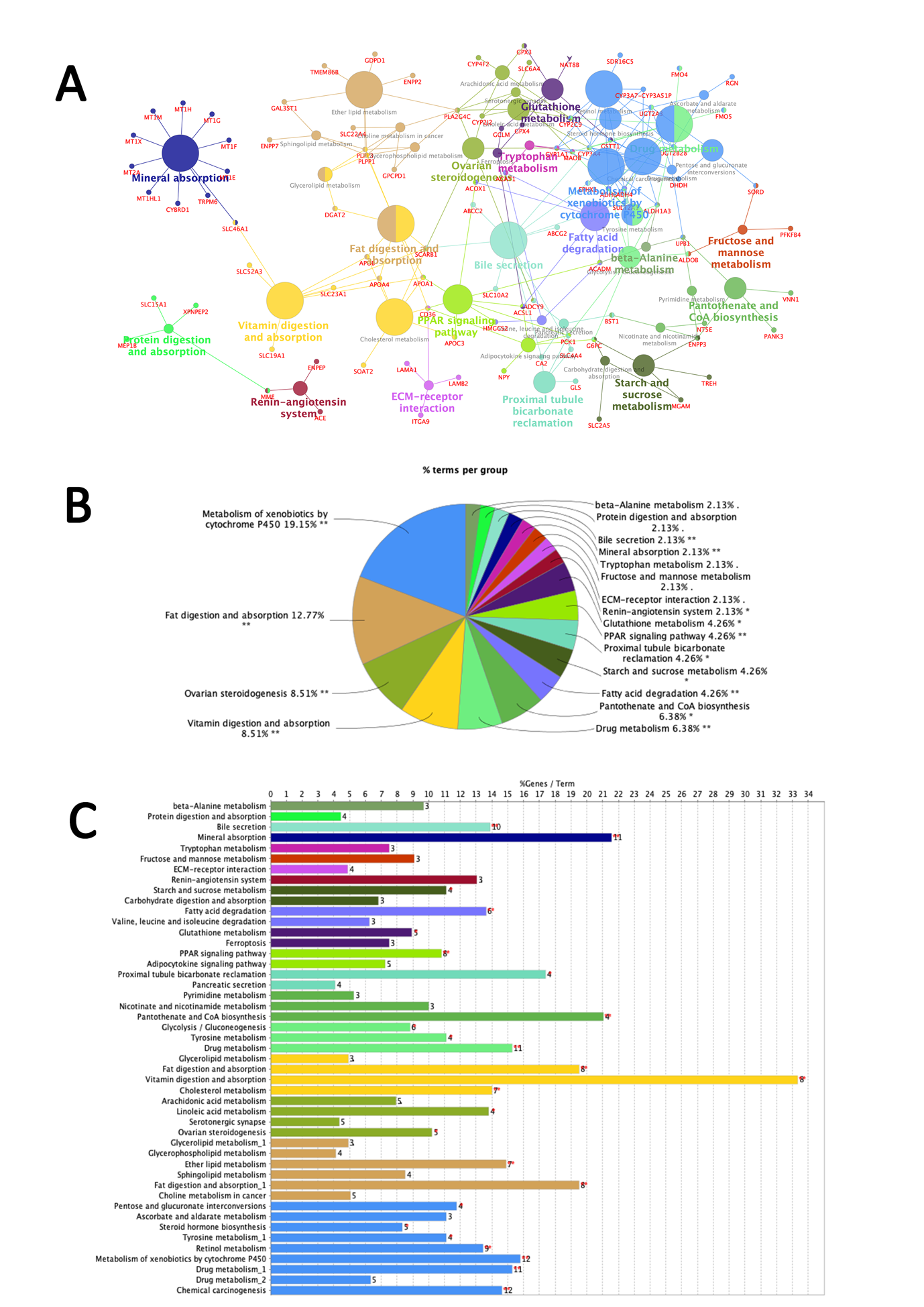


**Figure S9: KEGG pathways identified from downregulated PPI.**

A) Gene network interactions. B& C) Genes involvement in KEGG pathways in percentage and count. (A-C Figures generated using https://cytoscape.org/)


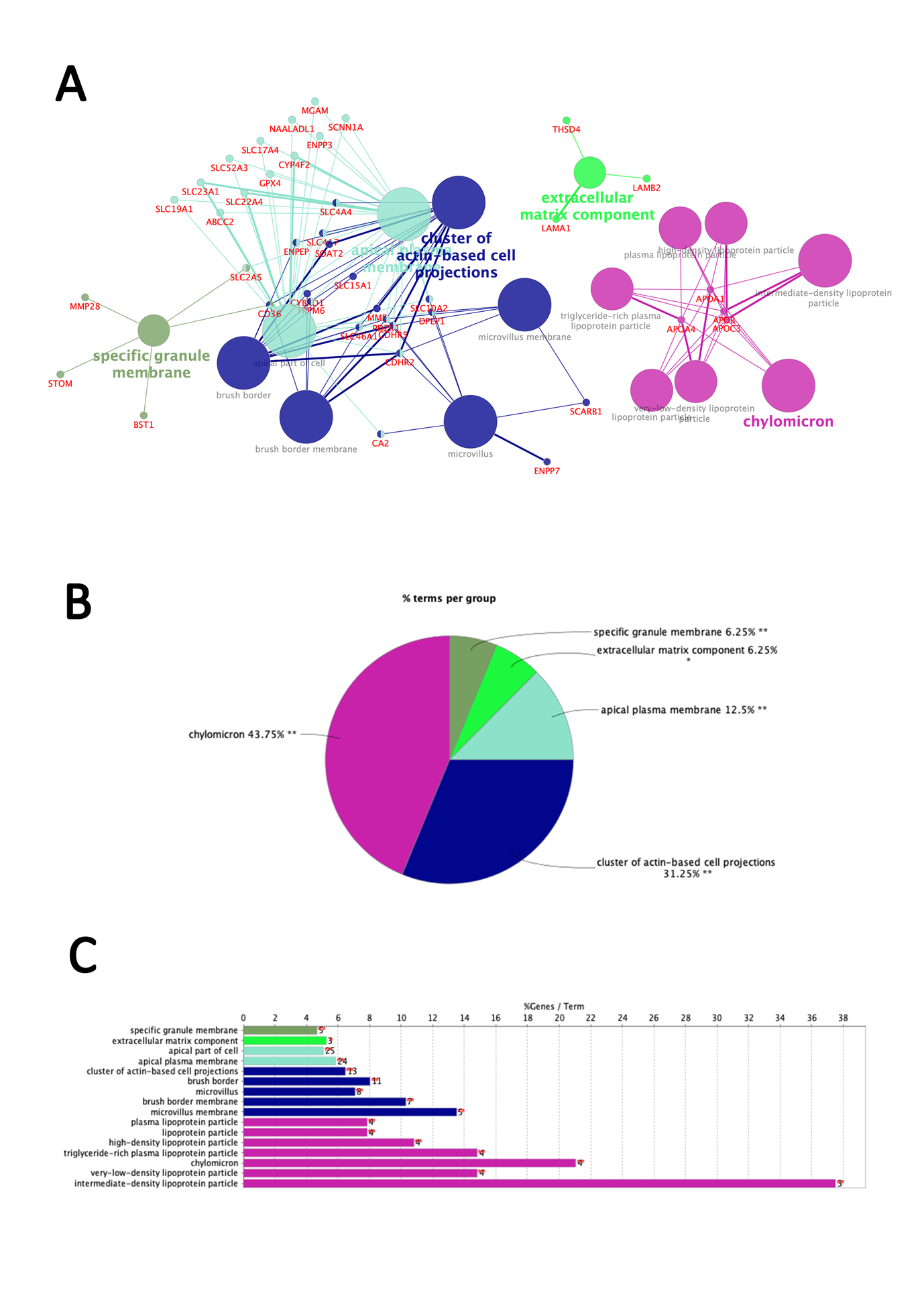


**Figure S10: Cellular components identified from downregulated PPI.**

A) Gene network interactions. B& C) Genes involvement in cellular components in percentage and count. (A-C Figures generated using https://cytoscape.org/)
